# Uncertainty quantification, propagation and characterization by Bayesian analysis combined with global sensitivity analysis applied to dynamical intracellular pathway models

**DOI:** 10.1093/bioinformatics/bty607

**Published:** 2018-07-13

**Authors:** Olivia Eriksson, Alexandra Jauhiainen, Sara Maad Sasane, Andrei Kramer, Anu G Nair, Carolina Sartorius, Jeanette Hellgren Kotaleski

**Affiliations:** 1 Science for Life Laboratory, Electrical Engineering and Computer Science, KTH Royal Institute of Technology, Stockholm, Sweden; 2 Science for Life Laboratory, Department of Numerical Analysis and Computer Science, Stockholm University, Stockholm, Sweden; 3 Swedish e-Science Research Centre (SeRC), KTH Royal Institute of Technology, Stockholm, Sweden; 4 Biometrics, Early Clinical Development, IMED Biotech Unit, AstraZeneca, Gothenburg, Sweden; 5 Centre for Mathematical Sciences, Lund University, Lund, Sweden

## Abstract

**Motivation:**

Dynamical models describing intracellular phenomena are increasing in size and complexity as more information is obtained from experiments. These models are often over-parameterized with respect to the quantitative data used for parameter estimation, resulting in uncertainty in the individual parameter estimates as well as in the predictions made from the model. Here we combine Bayesian analysis with global sensitivity analysis (GSA) in order to give better informed predictions; to point out weaker parts of the model that are important targets for further experiments, as well as to give guidance on parameters that are essential in distinguishing different qualitative output behaviours.

**Results:**

We used approximate Bayesian computation (ABC) to estimate the model parameters from experimental data, as well as to quantify the uncertainty in this estimation (inverse uncertainty quantification), resulting in a *posterior distribution* for the parameters. This parameter uncertainty was next propagated to a corresponding uncertainty in the predictions (forward uncertainty propagation), and a GSA was performed on the predictions using the posterior distribution as the possible values for the parameters. This methodology was applied on a relatively large model relevant for synaptic plasticity, using experimental data from several sources. We could hereby point out those parameters that by themselves have the largest contribution to the uncertainty of the prediction as well as identify parameters important to separate between qualitatively different predictions. This approach is useful both for experimental design as well as model building.

**Availability and implementation:**

Source code is freely available at https://github.com/alexjau/uqsa.

**Supplementary information:**

Supplementary data are available at *Bioinformatics* online.

## 1 Introduction

Dynamical models describing intracellular phenomena, like the protein interactions of signalling pathways, are increasing in size and complexity as more information from experiments is incorporated. These models are built from qualitative knowledge about the interaction topology, inferred from experiments like e.g. gene knock-outs, as well as from experimental quantitative data describing the input-output relationship of the observed system (Le Novere, 2015). The quantitative data are often sparse as compared to the size of the system, and trying to estimate parameters based on this data often results in large uncertainty in the parameter values, or that some parameters cannot be constrained at all given the data and model [i.e. are unidentifiable (Raue *et al.*, 2009)]. Parameter estimation from data (model calibration) rarely leads to precise point estimates for the parameters. Rather, the calibration often gives possible ranges for the parameters, and hence it is useful to provide distributions for the parameters, rather than to focus on single point estimates, i.e. to quantify the uncertainty in the parameter estimates (Vanlier *et al.*, 2013). Of interest is also to investigate how the uncertainty in the model parameters is transferred into uncertainty for predictions from the model, and to study how this uncertainty in the predictions can be mapped back and attributed to the different model parameters.

In this paper we develop and combine established methods for Bayesian inference and global sensitivity analysis (GSA) to show that they, when applied together to a relatively large and complex dynamical system involved in synaptic plasticity, give a comprehensive evaluation of the system given an experimental context, and can guide further experiments and modelling. Uncertainty analysis and GSA have often been performed as separate methods in different modelling studies, but here they are combined so that the GSA is performed based on the posterior distribution of the parameters and we consider system behaviors for which we have no data (i.e. predictions). The sensitivity analysis thereby reveals which parts of the model that are most unconstrained given a certain prediction. We can also compare different hypotheses and draw conclusions about parameters important for a certain model output.

### 1.1 Problem statement

**Fig. 1.**
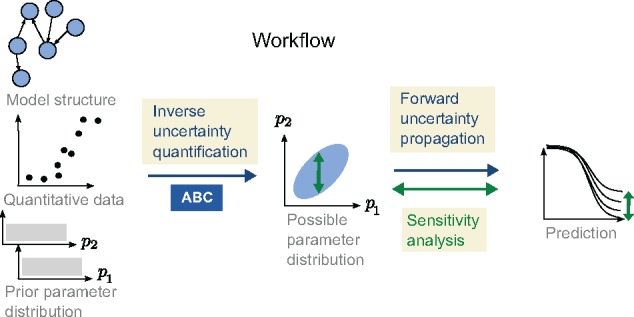
An illustration showing the different parts of the workflow and how GSA is applied on the prediction using the posterior distribution as a restriction on possible model parameter values

We start from a mathematical model, experimental data and a prior distribution of the parameters describing the prior knowledge (if any), see Figure 1. The model is described by the nonlinear system:

(1) $$\begin{array}{cc} & \dot{x}(t)=f(x(t),u(t),p)\\ & x(t_{0})=x_{0}\\ & y(t)=g(x(t),s) \end{array}$$

where x(t) corresponds to internal state variables (like protein concentrations in an intracellular model), u(t) to external input (e.g. an external signal to the cell, or the total amount of a specific protein), y(t) are the outputs, i.e. the observed variables (modelling counterparts to possible experimental readouts), ***p*** are system parameters (e.g. kinetic rate constants) and ***s*** are parameters for the readouts, like scaling factors. It can be noted that the parameters θ=(p,s) together with the initial conditions $x(t_{0})$ and the input u(t) fully specify the output from the system.

When experimental data are available corresponding to all or a subset of the system outputs, we denote these data $y_{w}^{\;\text{exp}\;}$, where the index *w* indicates a specific experimental setup (for details see Supplementary Section S1). The corresponding simulated data points from the model (under the same setup) are denoted $y_{w}^{sim}$. Within this study we only consider steady state output, or output at one specific time point, and therefore from here on we leave out indication of time in the notation. If there are output variables for which we do not have any corresponding experimental data, we denote them $y_{w}^{\text{pred}}$.

The problem we would like to address is to describe the uncertainty in the predicted output $y^{\text{pred}}$ given the model (1), the data $y^{\;\text{exp}\;}$, and the prior knowledge we have on the parameters (we drop the index *w* for ease of notation). We would also like to map out those parameters that contribute the most to the prediction uncertainty, as well as those parameters which are important in order to produce qualitatively different predictions (here corresponding to different types of plasticity). To achieve this, we consider the parameters θ to be stochastic variables (large letters will be used for stochastic variables, e.g. Θ) and we use a three step workflow, as illustrated in Figure 1. The workflow consists of (i) inverse uncertainty quantification, (ii) forward uncertainty propagation and (iii) GSA.

## 2 Background and existing methods

The purpose of **inverse uncertainty quantification** is to estimate unknown parameters of a model from observed data, and at the same time quantify the uncertainty in these parameter estimates. In a Bayesian framework, this is most often done (see for example Calderhead and Girolami, 2011; Kramer *et al.*, 2010; Toni *et al.*, 2009) by characterizing the posterior distribution, $f_{\Theta |Y^{\;\text{exp}\;}}(\theta |y^{\;\text{exp}\;})$, of the parameters. Here $Y^{\;\text{exp}\;}$ and Θ are the stochastic variables corresponding to the experimental data and the parameters, respectively, but for ease of notation we will drop the subscript and refer to the posterior as $f(\theta |y^{\;\text{exp}\;})$. The posterior distribution describes the uncertainty in a set of parameters of a specific model given observed data. The posterior distribution can, by the use of Bayes law, be deduced from the data likelihood $f(y^{\;\text{exp}\;}|\theta )$, which describes the likelihood of observing the data $y^{\;\text{exp}\;}$ from the model given that the parameters θ are used, and a prior distribution f(θ), describing the prior knowledge you have about the parameters. The posterior distribution corresponds to

(2) $$f(\theta |y^{\;\text{exp}\;})=\frac{f(y^{\;\text{exp}\;},\theta )}{f(y^{\;\text{exp}\;})}=\frac{f(y^{\;\text{exp}\;}|\theta )f(\theta )}{f(y^{\;\text{exp}\;})}$$

Often the posterior distribution cannot be expressed analytically, rather a sample from the distribution has to be retrieved in order to characterize it. In most cases this is done by the use of Markov chain Monte Carlo (MCMC) methods (Gelman *et al.*, 2013). Furthermore, the standard Bayesian framework is likelihood based, in the sense that we can deduce and compute the data likelihood $f(y^{\;\text{exp}\;}|\theta )$. When this is not the case, it is possible to turn to Approximate Bayesian Computation (ABC) (Marjoram *et al.*, 2003; Sunnåker *et al.*, 2013; Toni *et al.*, 2009) which relies on simulation followed by a comparison of simulated and experimental data to assess model fit. In ABC, samples from a prior distribution (or a proposal distribution) are accepted if the experimental data are reproduced by simulations from the model within a certain margin, so that a distance measure $\rho (S(y^{\mathrm{sim}\;\;}),S(y^{\;\text{exp}\;}))$ is smaller than some predefined cut-off *δ* (*S* is a summary statistic of the data). The accepted parameter sets θ will form the approximate posterior distribution $f(\theta |\rho (S(y^{\;\text{exp}\;}),S(y^{\mathrm{sim}\;\;}))\leq \delta )$. ABC can be used either together with MCMC or with simple rejection sampling.

The parameter space corresponding to the uncertainty in the parameters is related to what is also referred to as the viable parameter space of a system (Zamora-Sillero *et al.*, 2011), i.e. the subset of the parameter space where a model contains a desirable behaviour. Further approaches to explore the viable space have been described in the literature. In e.g. Gomez-Cabrero *et al.* (2011) particle swarm optimization is used to investigate the viable space.

The extent to which it is possible to deduce values of model parameters via inverse quantification is connected to the identifiability of the parameters. If the true values of the parameters can be deduced from unlimited data, the model is called identifiable. In Raue *et al.* (2009), identifiability is explored via the so called profile likelihood, and in Vanlier *et al.* (2012b), the profile likelihood methodology is integrated with a Bayesian approach to deal with non-identifiability.

**Forward uncertainty propagation and global sensitivity analysis** The uncertainty in the model parameters can be propagated to the model predictions, and here be quantified by e.g. the variance of the predictions at a specific time point or steady state. It is of interest to see how this uncertainty in the predictions depends on the uncertainty of specific parameters; i.e. to perform a GSA on the predictions based on the posterior distribution. It is not necessarily the case that an uncertain parameter will give uncertain predictions (Gutenkunst *et al.*, 2007). In general when performing GSA, the input factors (e.g. model parameters) are assumed to be independent and the GSA is then performed by sampling the factors independently from some marginal distributions (Saltelli *et al.*, 2008). Subsequently, the sensitivities are calculated by e.g. decomposing the output variance based on subgroups of input factors (Saltelli, 2002; Sobol, 2001). Dependencies between parameters make GSA more complex. Methods based on the decomposition of variances can still be used, but the calculation of sensitivities are more expensive and harder to interpret (Saltelli *et al.*, 2004). Another approach is to use so called Monte Carlo filtering, in which the output is subdivided into different classes and the respective parameter distributions are compared (Saltelli *et al.*, 2004). Other methods, some based on information theory, have also been presented in different studies (Lüdtke *et al.*, 2008; Vanlier *et al.*, 2012a). More methods for different forms of GSA are reviewed in e.g. Zi (2011).

## 3 Approach

The approach presented here combines Approximate Bayesian Computation for the inverse uncertainty quantification with decomposition of variance and Monte Carlo filtering for the GSA (Fig. 1). We have made some developments to the standard implementations of these methods in order to be able to combine them as well as to make the workflow more efficient, as discussed below.

**Inverse uncertainty quantification through ABC and efficient merging of data** The first step of the workflow consists of characterizing the posterior distribution of the parameters. In order to avoid assumptions of a normal likelihood we use simulation with ABC to sample from the posterior distribution, as non-normal output distributions easily can arise in non-linear systems (Weiße *et al.*, 2010).

**Fig. 2.**
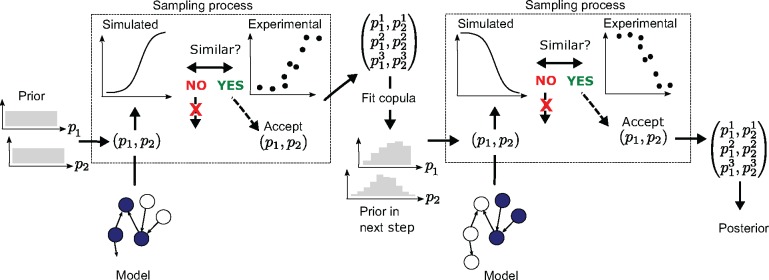
Sequential approach for mapping out the viable space when multiple experimental datasets corresponding to different experimental setups are available. In this case we have two experimental setups available (used in each of the dashed boxes and exemplified by different parts of the model being active as indicated by filled circles), and exemplify the approach for two parameters. The model calibration is done in steps, so that we start with a uniform prior for the first dataset, which produces a posterior distribution used as the prior in the next step by fitting and sampling from a multivariate copula model of the distribution. The process shown within the dashed boxes (draw from prior, simulate data, compare to experimental data and keep/not keep parameter set) is repeated many times using MCMC

We use several experimental datasets that are combined in sequence, where the posterior distribution after fitting to one dataset is used as the prior for the fitting to the next, by means of multivariate distributions called copulas (see below and Fig. 2). A Markov Chain Monte Carlo (MCMC) approach is used for the ABC sampling (ABC-MCMC) on each dataset in the sequence. In each ABC-MCMC iteration, we use an adaptive acceptance threshold (or margin) to more efficiently find the viable space where the actual sampling can begin. This is similar to the particle approach proposed by Secrier *et al.* (2009) where the acceptance region is decreased in consecutive runs, although we make this adaption within a single MCMC run.

Copulas are multivariate probability distributions with uniform marginal distributions, which describe the dependence structure between the stochastic variables. Graphical models called vines can be used to formulate copulas that are constructed in pairs in order to describe the dependencies over multiple variables (Bedford and Cooke, 2002) (see also further details in the Supplementary Material). We use R-vines to model the multivariate posterior distributions produced by ABC-MCMC runs. After each step of the fitting sequence described above, a copula is fitted to the posterior sample from that step and is next used as prior for the subsequent step. To our knowledge, copulas have not been employed in this way in inverse quantification previously, although they have been used in hybrid proposal distributions in MCMC (Schmidl *et al.*, 2013). The proposed approach to inverse uncertainty quantification is illustrated in Figure 2 and described in pseudocode in Supplementary Section S2.5. A validation of the approach is also presented in Supplementary Section S2.6. The inverse quantification methodology was implemented in R with the use of the VineCopula package (Schepsmeier *et al.*, 2018).

Based on the posterior distribution, we characterize to what extent different parameters are constrained by the data and model by the entropy of the marginal distributions. The entropy was approximated from the normalized sample histograms and calculated by $H\;=-\sum_{k=1}^{n}p_{k}\;\;\text{ln}\;(p_{k})\Delta b$, where $p_{k}\;$ is the marginal probability density value in the *k*:th bin, Δb is the bin width and *n* the number of bins. The reduction in entropy observed when updating the parameter distributions from the prior to the posterior is used as a measure of the uncertainty decrease of the specific parameters, $H_{\text{diff}}=H_{\text{prior}}-H_{\text{post}}$. The posterior distribution is further characterized by different standard statistical tools like clustered correlation plots and parallel coordinate plots.

**Forward uncertainty propagation and global sensitivity analysis** The next step of the workflow is to translate the uncertainty in the parameters to uncertainty in predictions by performing simulations based on all parameter sets in the posterior distribution sample. The uncertainty of the predictions $Y^{\text{pred}}$ is next quantified by the variance of each vector element $V(Y^{\text{pred}})$.

Finally, we perform a GSA to investigate from where the uncertainty in the prediction stems. This is done in two ways with two different aims. First, we investigate which parameters on average reduce the uncertainty in the prediction the most if they were known more precisely. Second, we look into which parameters are most influential in separating different qualitative behaviours of the model.

In order to address the first aim we decompose the variance of the output based on the contribution from different input factors of the model (model parameters in our case). The first order sensitivity index (Saltelli *et al.*, 2004) quantifies the impact that a model parameter has on a specific output, and is defined by $S_{i}=V_{\Theta_{i}}(E_{\Theta_{-i}}(Y^{\text{pred}}|\Theta_{i}))/V(Y^{\text{pred}})$. Here $\Theta_{-i}$ stands for all parameters of the vector Θ, except Θ_*i*_. The expression $E_{\Theta_{-i}}(Y^{\text{pred}}|\Theta_{i})$ is thus the expected value of $Y^{\text{pred}}$ over all parameters except Θ_*i*_ when Θ_*i*_ is conditioned on a specific value $\Theta_{i}=\theta_{i}^{*}$, and $V_{\Theta_{i}}(\ldots )$ is the variance over all specific values $\theta_{i}^{*}$. Well established and efficient methods to calculate *S_i_* for distributions of independent input factors (Saltelli, 2002; Sobol, 2001) are available. However, since we are performing the GSA using the multivariate posterior distribution, $f(\theta |y^{\;\text{exp}\;})$, which displays dependencies between possible model parameter values due to the inner structure of the model, these methods cannot readily be applied. Instead, we perform a calculation inspired by [Saltelli *et al.* (2004), chapter 5.10], but with modifications in order to utilize the already existing posterior sample produced from the ABC method. This computation is based on binning the posterior space and results in an approximation of the sensitivity index *S_i_* (details can be found in the Supplementary Material).

If the model is not sufficiently constrained by the experimental data, a large variance can be seen in the prediction and qualitatively different output behaviours can be observed. It is then of interest to identify the parameters with the largest impact in separating these behaviors. This is known as Monte Carlo filtering (Saltelli *et al.*, 2004). In order to do this we first group the predictions into classes with different qualitative behaviour, and also divide the posterior distribution sample according to the same grouping. Model parameters that have a large influence on the model behavior in question display different sample distributions in the different groups. We consider marginal as well as pairwise parameter distributions, and sort them based on the Kolmogorov-Smirnov test and Kullback-Leibler divergence, respectively. The GSA methodology was implemented using MATLAB.

## 4 Application

**Fig. 3.**
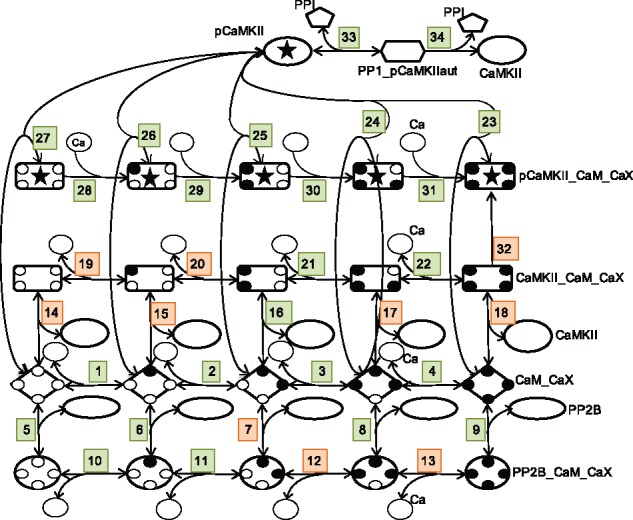
A graphical representation of the intracellular model used to illustrate the proposed approach to model characterization. The numbers indicate the corresponding reactions in Supplementary Table S2. The small black filled circles correspond to Ca domains that have bound a Ca and the X in CaX correspondingly denotes Ca,…, Ca4 (Ca0 is simply referred to as Ca in Supplementary Table S2). Reactions colored in orange correspond to parameters that would be the best targets to identify in order to reduce prediction uncertainty, and correspond to the parameters in the legend of Figure 6

We have applied our approach to a previously constructed intracellular model that in a simplified way exemplifies a molecular mechanism important for the strengthening (long term potentiation, LTP) or weakening (long term depression, LTD) of neuronal synapses (Nair *et al.*, 2014). The modification of synapses through the process of LTP or LTD is a complicated process including a number of kinases, phosphatases and scaffolding proteins (Woolfrey and Dell'Acqua, 2015). This process is, however, often assumed to be effectuated by the balance between a few important kinase and phosphatase enzymes, and in the model used in this study (Nair *et al.*, 2014), this balance is due to the interaction between calcium (Ca), calmodulin (CaM), which contains four Ca-binding domains, protein phosphatase 2B (PP2B, also known as Calcineurin), Ca/CaM-dependent protein kinase II (CaMKII) and protein phosphatase 1 (PP1), as illustrated in Figure 3.

### 4.1 Model

The model consists of 25 species (corresponding to proteins, protein complexes, the activated form of a protein or Calcium) and 34 reactions, where all reactions except two are elementary reversible reactions based on the law of mass action. This means that the reactions are of the type: A+B⇄*C*, where *A*, *B* and *C* are different species, where the right going reaction has a kinetic constant denoted *k_f_* and the reaction in the opposite direction has a kinetic constant denoted *k_r_*. We also use the equilibrium constants $K_{d}=k_{r}/k_{f}$. All species and reactions are listed in Supplementary Tables S1 and S2, respectively. There are also thermodynamic constraints which apply when there is more than one reaction path between a pair of species. These are expressed by the so called Wegscheider conditions (Gorban and Yablonsky, 2011; Wegscheider, 1911; Yablonskii, 1991) and link some *K_d_* parameters of the model to other *K_d_* parameters (Supplementary Table S3). We therefore decompose the *K_d_*-parameters in the model into two sets; free *K_d_*-parameters that are modified throughout the analysis, and thermo-constrained *K_d_*-parameters whose values are set by the values of the free parameters via these rules. More information about the model can be found in Supplementary Section S1.

### 4.2 Experimental data for parameter estimation

The parameter estimation was based on quantitative data collected from a number of publications (Bradshaw *et al.*, 2003; O'Donnell *et al.*, 2011; Shifman *et al.*, 2006; Stemmer and Klee, 1994) as described in Nair *et al.* (2014). The data correspond to different experimental setups describing different, experimentally engineered, phenotypes of the system. The phenotypes correspond to a subpart of the system, i.e. only a subset of the species was used in that experiment, e.g. only CaM and Calcium for phenotype 1 (see Supplementary Table S2). Each phenotype is characterized by steady state (or close to steady state) input-output curves, i.e. in each experiment, a given input (like [Ca]) is varied in value in order to obtain the curve for the output (e.g. Mol bound Ca per Mol CaM). In the model, the experimental phenotypes are recreated by applying different model inputs ***u***. The different phenotypes, and the subparts of the model that are active under the different settings, are described in detail in Supplementary Section S1 and Supplementary Table S2.

### 4.3 Prior distributions

We obtained default values for the free parameters from Nair *et al.* (2014), with a few updates, and used them as the centers *μ_i_* for log-uniform prior distributions. The range of the prior was set as $\mu_{i}-3$ to $\mu_{i}+3$ in log-space. We did not sample the thermodynamically constrained parameters, nevertheless, we can assign implicit prior distributions to them via the thermodynamic constraint rules (Supplementary Table S3). By sampling the free parameters and propagating these through the rules, we can obtain prior samples for the constrained parameters as is shown in Supplementary Figure S8.

### 4.4 Model reduction

The different phenotypes correspond to situations either very close to steady state or with slow dynamics, and we have utilized this fact in order to reduce the model. For some of the phenotypes (phenotypes 1–4 in Supplementary Table S2) the output could be approximated with steady state. Steady state reduction was hence performed to the model in order to speed up calculations, resulting in analytical steady state solutions for subparts of the model. The reduction was based on the principle of detailed balance (Yablonskii, 1991), which has the consequence that steady state only can occur at an equilibrium and thereby all reaction fluxes are zero. Since the reaction fluxes are of the form $k_{f}[A][B]-k_{r}[C]$, it follows that the equilibrium concentrations of the species depend only on $k_{r}/k_{f}=K_{d}$ (using that the equilibrium equations can be rewritten as $\text{log}[A]+\text{log}[B]-\text{log}[C]=\text{log}(k_{r}/k_{f})=\text{log}(K_{d})$. In this way, the equilibrium equations were solved analytically, while making use of the mass conservation laws of the system (i.e. that the total amount of each elementary species remains the same during the experiment). This enabled us to express the equilibrium concentrations as functions of the *K_d_* parameters and the total amounts of the species. More information about the analytical solutions can be found in the Supplementary Section S7.

For the remaining phenotypes (phenotypes 5–6 in Supplementary Table S2) we utilized the fact that they have semi-steady state dynamics, and that the output therefore mainly should depend on the *K_d_* parameters. The problem was thereby reduced to first finding the posterior for the *K_d_* parameters, based on constant *k_f_*, and then expand this posterior to *k_f_* : s by simple rejection sampling.

### 4.5 Results

**Inverse uncertainty quantification and characterization of the viable space** Given the prior distributions, experimental data and model structure, a sample from the posterior distribution was retrieved through the sequential ABC-method that had a good fit to the experimental data (details on the distance measure and normalization procedures used can be found in the Supplementary Material). The sampling was performed on a parameter log-scale and the multivariate posterior distribution was characterized by looking at single parameters as well as pairs of parameters.

**Fig. 4.**
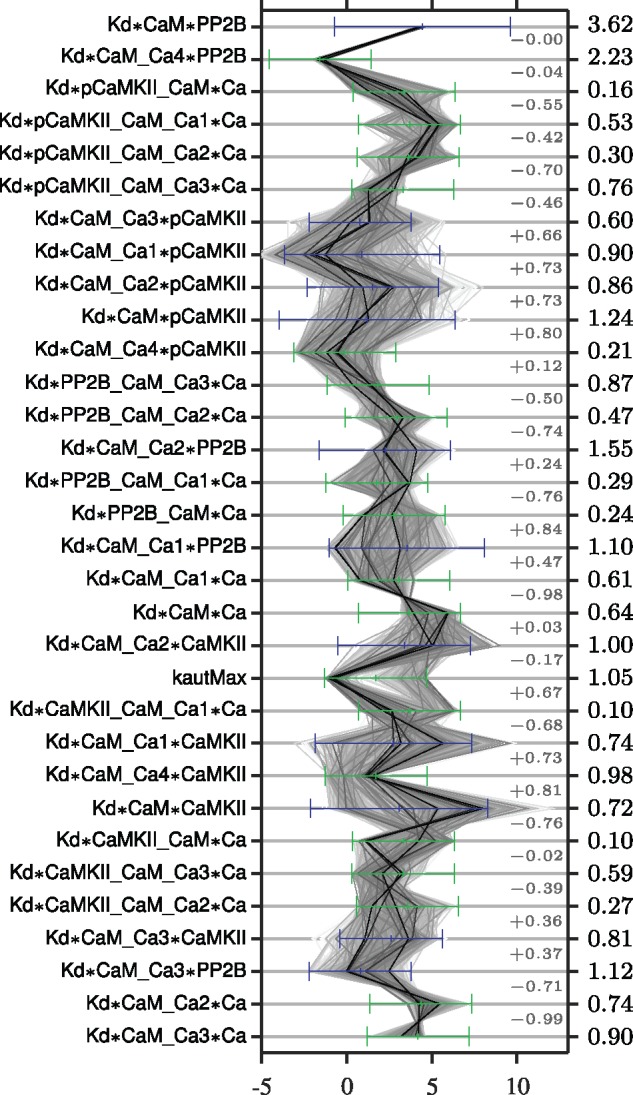
Illustration of the marginal posterior distribution and reduction in entropy for all parameters. The numbers indicated to the far right correspond to the reduction in entropy ($H_{\text{diff}}$) when going from prior to posterior distribution, and the light grey numbers correspond to the pairwise correlations. Each sample in the posterior distribution is connected across the parameters by a thin grey line, the darkness of which reflects to the posterior probability density at that sample point (the kernel density estimate). The prior of the free parameters is indicated by green bars (showing the range of the log-uniform distribution), and the prior of the thermo-constrained parameters (calculated through the equations of Supplementary Table S3) is indicated by blue bars (showing one standard deviation of the, lognormal-looking, distributions)

The marginal posterior distributions of all *K_d_* parameters are summarized in the parallel coordinate plot of Figure 4, where the prior distribution and reduction in entropy also are indicated. The forward *k_f_* and backward *k_r_* parameters are not included in the figure since these had, as expected since we use mainly steady state data to fit the model, a posterior distribution very similar to the prior (and a corresponding low reduction in entropy). The parameters corresponding to reactions 33 and 34 of Figure 3 are also not included since this part of the model is only used for the prediction (see Supplementary Table S2). It can be noted that some parameters are very constrained by the model and currently used data, with the two most prominent examples being $K_{d}$*CaM*PP2B and $K_{d}$*CaM_Ca4*PP2B, which both have a narrow distribution and a large reduction in entropy. Other parameters instead occupy the parameter space up to the edge of the prior, e.g. $K_{d}$*CaMKII_CaM_Ca1*Ca and kautMax (see Section 5). This could be a sign of the prior being too small to include the full viable space or a sign of non-identifiability, which is (artificially) resolved by imposing a prior.

Most parameters that have a narrow posterior distribution (like $K_{d}$*CaM*PP2B) display a corresponding large reduction in entropy and vice versa. Parameters can however have a wide posterior distribution and at the same time have a large reduction in entropy, e.g. kautMax which displays a prominent bimodality in the marginal distributions (Fig. 4 and Supplementary Fig. S8). Bimodal distributions can contain a lot of information about the parameter, despite possibly having a wide spread. Histograms of the marginal posterior distributions and their characteristics, such as model-based credibility intervals, are given in Supplementary Figure S8 and Tables S4 and S5.

**Fig. 5.**
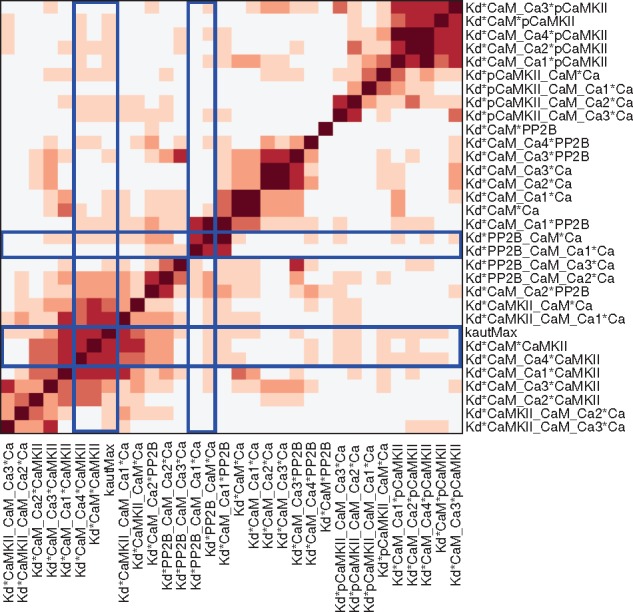
Correlation plot of model parameters based on the samples from the posterior distribution. The parameters are clustered based on their correlation profile, i.e. the array of correlation coefficients (using absolute values) for each parameter, via hierarchical clustering with an Euclidean distance metric and average linkage. The blue lines frame the arrays with correlations related to the bimodal parameters

We also examined possible couplings between parameters by a clustered correlation plot (Fig. 5), where the parameters are clustered into groups based on their correlation profile. Some parameter pairs show large correlations, while most others appear to be uncorrelated or only weakly correlated. The pattern of correlations between the parameters of the model also have a tendency to follow the model structure, so that parameters with a high value in the correlation plot are close in the graph of Figure 3. It can also be noted that the parameters with a bimodal distribution are clustered into two groups as illustrated by blue frames in Figure 5. This is probably an indication that if one parameter is bimodal this can result in bimodality of the correlated parameters as well.

**Forward uncertainty propagation** We next analyzed how the uncertainty in the parameters is propagated to uncertainty in the prediction that we would like to make from the model. The prediction used here to demonstrate the workflow corresponds to the relationship between the active form of the kinase, CaMKIIact, and the active form of the phosphatase, PP2Bact, and how this relationship depends on the frequency of Ca transients given as input [for details on input and output functions see Supplementary Section S1 and Nair *et al.* (2014)]. For higher frequency the Ca summates into increased amplitudes. The presence of a large amount of activated CaMKII relative to activated PP2B is assumed to give long term potentiation (LTP) with the reverse relationship instead resulting in long term depression (LTD).

**Fig. 6.**
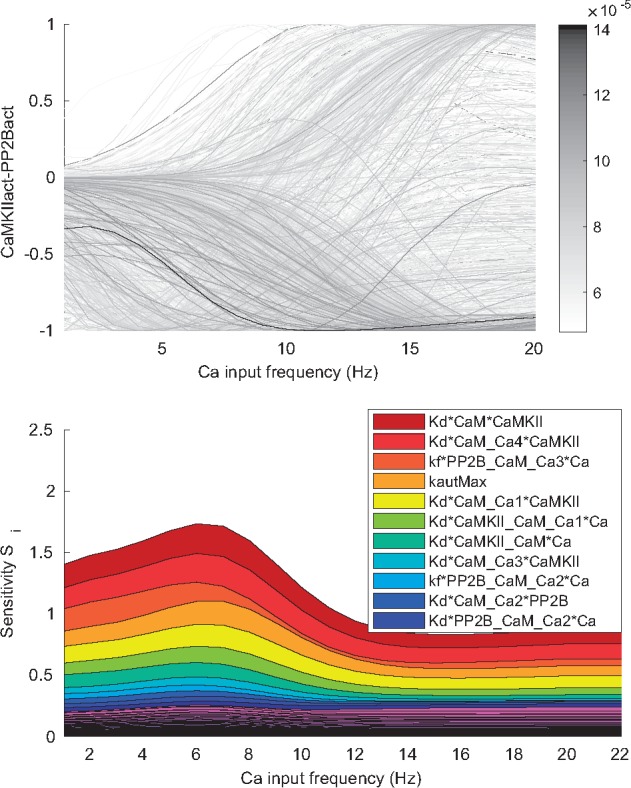
Uncertainty and sensitivity of the prediction. The prediction corresponds to the (normalized) difference between the activity of CaMKII and PP2B at different Ca frequencies. Top panel: The different outputs (grey lines) correspond to different sample points from the posterior distribution. A large uncertainty in the prediction can be observed. Bottom panel: First order sensitivity index (*S_i_*) for all parameters at different input (Ca frequency) values. Some parameters have a large influence on the uncertainty

For all parameter sets of the posterior distribution we calculated the corresponding CaMKIIact-PP2Bact relationship at different Ca frequencies (Fig. 6). There is a large variation in the prediction given a certain Ca frequency, showing that the model, with currently used data, is not sufficiently constrained to give a precise prediction of this behavior. In order to investigate the best way to reduce this uncertainty and to learn more about the system we next performed a GSA.

**Global sensitivity analysis** First we analyzed how the uncertainty in the different parameters contributes to the uncertainty of the prediction by decomposing the variance of the prediction based on the different single parameters (see Approach). The single parameters that, if known, on average would give the largest reduction in the uncertainty of the prediction are shown in the legend of Figure 6 and are also indicated in Figure 3. Dissociation constants corresponding to the binding of CaM to CaMKII (reactions 14, 15, 17 and 18 in Fig. 3 and Supplementary Table S2), as well as the CaMKII-CaM complex binding the first two Ca (reactions 19 and 20) are important. Also the maximal autophosphorylation rate (reaction 32), and CaM bound to PP2B binding the third and fourth Ca (reaction 12 and 13) would give a large reduction in uncertainty. It can be noted that several of these parameters are correlated within the posterior distribution (Fig. 5). This means that knowledge about one parameter would automatically decrease the uncertainty in the other correlated parameters as well. One possible approach for further investigation would therefore be to identify the most sensitive parameter of each cluster of correlated parameters and try to experimentally determine its value. The remaining parameters in each cluster would then likely also show a large decrease in uncertainty.

**Fig. 7.**
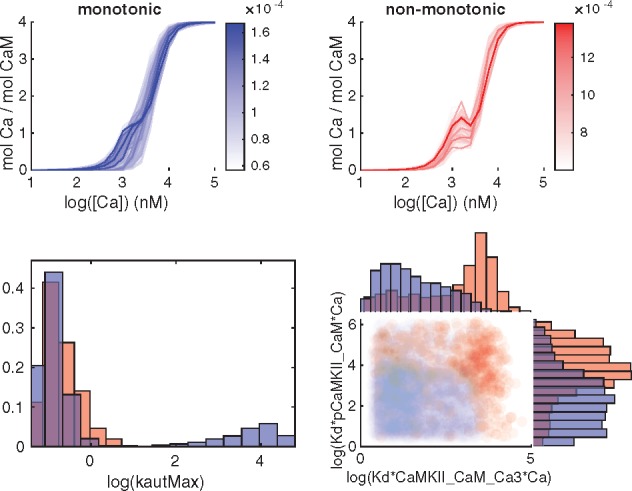
Classification of outputs from phenotype 5 and corresponding subdivision of the posterior distribution. Red corresponds to non-monotonous output while blue corresponds to the monotonous output curves. Top panel: Subdivision of the output mol Ca per mol CaM into a monotonous and non monotonous group. Bottom left panel: Marginal histograms for a parameter with a large difference in the posterior distribution between the two classes, as quantified by the Kolmogorov-Smirnov test. Bottom right: Pairwise scatterplots of the parameter pair with the largest KLD-distance

**Fig. 8.**
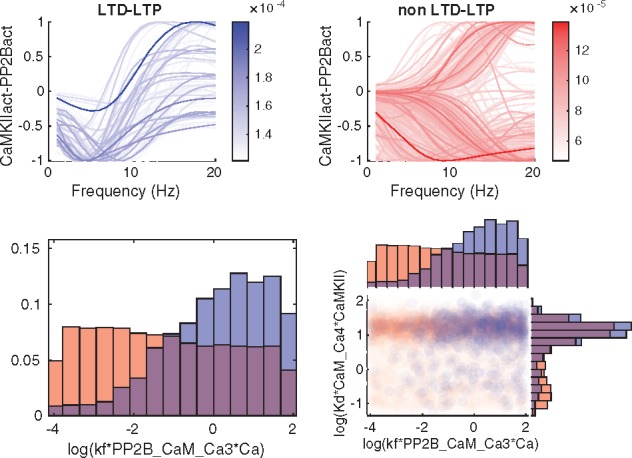
Classification of predictions and corresponding subdivision of the posterior distribution. Blue corresponds to the hypothesized output behaviour for synaptic plasticity (LTD-LTP) while red corresponds to the curves that do not follow the hypothesized behaviour (non LTD-LTP). Top panel: Subdivision of the prediction output according to the two classes. Bottom left panel: Marginal histograms for the the parameter with the largest difference in posterior distribution between the two classes quantified by a Kolmogorov-Smirnov test. Bottom right panel: Pairwise scatterplots for a high KLD-scoring pair

The next part of the GSA was to analyze qualitatively different types of output behaviors via Monte Carlo filtering. We show the output corresponding to the prediction as well as the output for phenotype 5 in Figures 7 and 8. Phenotype 5 corresponds to an experimental setting including CaM, CaMKII and Ca, with different levels of constant [Ca] as input and the amount of bounded Ca as output (Supplementary Table S2). It was included since it exhibits a somewhat peculiar behavior. Both outputs were divided into two different classes (Figs 7 and 8, top panels) depending on whether, in the case of phenotype 5, the output was monotonic or not, and for the prediction, whether or not the output agreed with a hypothesized behaviour for synaptic plasticity. The sample from the posterior distribution was also subdivided according to the same classes and analyzed both at the individual parameter level and by investigating all parameter pairs. (Figs 7 and 8, bottom panels). The individual parameters as well as the parameter pairs were sorted based on the distance between the distributions when comparing the two classes. A Kolmogorov-Smirnov test was used for the marginal distributions for the individual parameters, and the Kullback-Leibler divergence (KLD) was employed for the joint distribution of parameter pairs.

For phenotype 5, an interesting example is the parameter kautMax (Fig. 7, bottom left), corresponding to reaction 32. For large values of this parameter only monotonic output curves are observed, but for smaller values we have both types of curves. When pairs of parameters are considered, the scatterplot corresponding to the pair with the largest KLD distance, $K_{d}$*pCaMKII_CaM*Ca versus $K_{d}$*CaMKII_CaM_Ca3*Ca, shows an interesting separation between classes (Fig. 7, bottom right). Further histograms and two dimensional projections of top scoring parameters and pairs are shown in Supplementary Figures S9 and S11.

For the prediction, the hypothesized output behaviour for synaptic plasticity describes a specific relationship between Ca level and kinase-phosphatase activity (denoted CaMKIIact-PP2Bact, in Fig. 8). This relationship is as follows. At *very low levels* of Ca there is a balanced kinase-phosphatase activity, for *low levels* there is a negative balance (i.e. phosphatases dominate), and for *larger levels* of Ca there is a positive balance (i.e. kinases dominate). The classification criterion, which was based on whether this behavior is shown or not, is formalized mathematically in Supplementary Section S3.3.

Similarly, for this output we observe differences between the two classes with respect to the marginal parameter distributions (see Supplementary Fig. S10 for histograms of top scoring parameters), but for this output, the number of parameters with a clear separation between the two class distributions are much fewer than in the earlier case with phenotype 5. The bottom left panel of Figure 8 shows the histogram of the parameter with the largest distance between the classes. For $k_{f}$*PP2B_CaM_Ca3*Ca there is a larger probability of receiving the hypothesized behavior for higher values. Looking at the top scoring KLD-pairs (Supplementary Fig. S13) does not provide any extra information as compared to the histograms. One of the highest scoring pair is shown in the bottom right panel of Figure 8.

The goal of the GSA approach is first of all to give information about parameters that are important to restrict, in order to reduce the uncertainty in the prediction. Secondly, for parameters that we know little about, to evaluate how they affect the output. Using this approach, we will not obtain much information on possible biological mechanisms for parameters that are already well constrained. Nevertheless, it is interesting to see why different parameters show up in the sensitivity analysis, and we discuss possible mechanisms in some detail in the Supplementary Section S5.

## 5 Discussion

We have here presented a workflow for analyzing the viable space of biochemical models. We assume a certain model structure (i.e. the set of reactions) and a particular dataset for constraining the model. This workflow was tested on a previously constructed model of CaMKII and PP2B activation. By combining Bayesian analysis with GSA we can quantify the uncertainty in the model parameter estimates and model predictions, as well as pinpoint where this uncertainty stems from. This is useful both for experimental design as well as model building.

Biochemical models are generally uncertain (Geris and Gomez-Cabrero, 2016) with a large viable space. Performing an a-priori GSA, e.g. based on a product space of posterior intervals, would lead to model behavior far outside the bounds set by the data and lead to errors. When GSA is performed based on the posterior distribution it takes the correlations between parameters into account and only investigates data fitting parameters.

Analyzing the viable space of complex models with many parameters is, however, computationally expensive. By the use of model reduction as well as integrating datasets sequentially with copulas we could reduce the computational cost to the point where an extensive analysis could be performed. Using copulas in this setting means that we make an approximation of the posterior space. We have done a thorough study on how good this approximation is for the smaller part of the LTD-LTP model corresponding to phenotypes 1–3 (Supplementary Section S2.6). The approximation works well for most instances, but seems somewhat less accurate for parts of the posterior space with very low density, especially when a high density mode is present. If the posterior distribution for a parameter pair displays disjoint parts, it is likely advantageous to divide the parameter space for the parameter pair into different regions and fit a copula to the samples of each region separately.

A Bayesian approach together with GSA is of course more rigorous than a manual parameter search, since it accounts for the variability in parameter space. It thereby provides more accurate and extensive predictions. It also offers predictions on parameter regions (e.g. levels of kinetic constants) which are correlated with desired behaviours. An additional value is that the formulation of a prior distribution makes the model assumptions more explicit, which is more useful when sharing and comparing models than a single, seemingly working parameterization.

There are other workflows of model analysis described in the literature which e.g. focus on fast optimization and statistical classification and clustering techniques (Gomez-Cabrero *et al.*, 2011), while the procedures presented here focus on handling uncertainty quantification and propagation in a consistent statistical setting for all parts of the analysis. On the other hand, several similar, statistically embedded experiment design methods (e.g. Liepe *et al.*, 2013; Weber *et al.*, 2012) focus on maximizing information in planned experiments, whereas this analysis workflow assigns roles to model constituents and measures of importance to parameters.

We have chosen large prior bounds in order to capture as much of the models behaviours as possible and to avoid biases and assumptions. More narrow bounds are reported in literature (e.g. Pepke *et al.*, 2010; Stefan *et al.*, 2008) and we could have used this information to exclude parameter regions from the prior. It is not trivial, however, to directly include results of other groups parameter estimation efforts. The methodologies and model complexity often differ enough to make the mapping of parameters from literature models onto another model difficult and especially the notion of what a *range* is differs from one methodology to another. However, as a test case, we used some of the reported parameter ranges to filter our posterior sample retrospectively, see Supplementary Section S6, and performed a new sensitivity analysis. Filtering of parameters already determined not to be sensitive did, as expected, not have much of an effect in the retrospective analysis. When we instead restricted the most sensitive parameter to the literature range and repeated the sensitivity analysis within that range, there was a large change in the sensitivity profile, even though a large uncertainty in the prediction remained (see Supplementary Fig. S7).

Even though we used a large prior, some of the parameters had a marginal posterior distribution that reached the edge of the prior, with kautMax being the most prominent example. We tested a wider prior for this parameter, which resulted in a slightly wider posterior distribution beyond the original prior. The characteristics of the posterior distribution for kautMax, with two modes, were however similar under both priors.

We have so far only spoken about the viable space in terms of model uncertainty due to missing data. Another reason that a viable space is a better description than a single parameter vector is biological variability, because biological measurement techniques often target cell populations rather than single cells. Biochemical pathway models, on the other hand, often correspond to a generic individual cell or cell compartment. With a Bayesian approach it is possible to capture (smoothly) varying biological properties, even though it cannot distinguish between uncertainty due to missing data and biological variability.

## Supplementary Material

Supplementary Data
