# Supplementary material for: Uncertainty quantification, propagation and characterization by Bayesian analysis combined with global sensitivity analysis applied to dynamical intracellular pathway models
Source: Bioinformatics. 2018 Jul 13;35(2):284–92. doi: 10.1093/bioinformatics/bty607 (PMC6330009; doi:10.1093/bioinformatics/bty607)
Supplement: Supplementary Data [file bty607_supplementary_data.pdf]

Supplement to:

Uncertainty quantification, propagation and characterization by Bayesian analysis combined with global sensitivity analysis applied to dynamical intracellular pathway models

Olivia Eriksson, Alexandra Jauhiainen, Sara Maad Sasane, Andrei Kramer, Anu G Nair, Carolina Sartorius, and Jeanette Hellgren Kotaleski

S1 Model details

S1.1 Additional information on the LTP LTD pathway - autophosphorylation

As described in the main text, the following elementary species are included in the model: calcium (Ca), calmodulin (CaM), protein phosphatase 2B (PP2B) and Ca/CaM-dependent protein kinase II (CaMKII) and protein phosphatase 1 (PP1). CaM is a Ca-binding protein involved in multiple signaling processes and is strongly implicated in synaptic plasticity. CaM contains four Ca-binding domains, each binding one Ca ion. The binding of Ca by CaM is a cooperative process. Ca-bound CaM activates PP2B, another protein implicated in molecular processes related to learning which also plays a role in striatal signaling. The third protein, CaMKII, is a kinase, which is activated by the binding of Ca-CaM. CaMKII molecules exist as dodecamers, consisting of two hexamer rings. A CaMKII unit that has bound CaM can autophosphorylate when sitting beside an active neighbouring unit in the same hexamer ring. The phosphorylated unit can remain active even in the absence of Ca-CaM.

S1.2 Additional information about the model reactions 32 (autophosphorylation) and 34

Two of the model reactions are not elementary reversible reactions of the type described in the main text; one of these is different only in the way that it is irreversible (reaction 34 in Table S2) , the other one is more complicated as it describes the autophosphorylation process of CaMKII monomers.This process is for practical reasons reduced with the help of a phenomenological rate function, and corresponds to reaction 32 in Table S2. The rate function $f_{\text{aut}}(x) = k_{\text{autMax}} g_{\text{aut}}(x)$ describes how much active CaMKII units that are autophosphorylated each time step as a function of the proportion of active CaMKII monomers, x. It consists of a constant k_{autMax} corresponding to the maximum rate, times a function

$$g_{\text{aut}}(x) = 3.90 \frac{x^2}{1 + 2.87x} \tag{S1}$$

describing the probability that an activated CaMKII monomer has another activated monomer as a neighbour (Li *et al.*, 2012). The constant values within the function g_{aut} were retrieved by fitting to the data of Figure S3 in Li *et al.*, 2012.

S1.3 Model inputs $\boldsymbol{u}$, outputs $\boldsymbol{y}$, initial conditions $\boldsymbol{x}_0$ and experimental conditions (phenoptypes)

The model is described in equation (1) in the main text. There the state $\boldsymbol{x}(t)$ is a vector corresponding to all model species listed in Table S1 (in that same order). The input $\boldsymbol{u}$ corresponds to the vector of input functions $\boldsymbol{u} = ([\text{Ca}](t), \text{CaM}_{\text{tot}}, \text{PP2B}_{\text{tot}}, \text{CaMKII}_{\text{tot}}, \text{PP1}_{\text{tot}})'$, where $[\text{Ca}](t)$ is either constant in time or corresponds to a spike train consisting of 10 spikes with frequency f, initiated after 30s (for a detailed description of the spike train, see Nair *et al.*, 2014 Figure 12.3 and Figure 12.4), the variables CaM_{tot}, PP2B_{tot}, $\text{CaMKII}_{\text{tot}}$ and PP1_{tot} are the (time conserved) total amounts of the respective species given by

$$\begin{aligned}
\text{CaM}_{\text{tot}} &= (0,1,0,0,0,1,1,1,1,1,1,1,1,1,1,1,1,1,1,1,1,1,1,1,0)\boldsymbol{x} \\
\text{PP2B}_{\text{tot}} &= (0,0,1,0,0,0,0,0,0,1,1,1,1,1,0,0,0,0,0,0,0,0,0,0,0)\boldsymbol{x}, \\
\text{CaMKII}_{\text{tot}} &= (0,0,0,1,1,0,0,0,0,0,0,0,0,0,1,1,1,1,1,1,1,1,1,1,0)\boldsymbol{x}, \\
\text{PP1}_{\text{tot}} &= (0,0,0,0,0,0,0,0,0,0,0,0,0,0,0,0,0,0,0,0,0,0,0,0,1)\boldsymbol{x}
\end{aligned}$$

Further, let

$$\begin{aligned}
\text{Ca}_{\text{bound}} &= (0,0,0,0,0,1,2,3,4,0,1,2,3,4,0,1,2,3,4,0,1,2,3,4,0)\boldsymbol{x}, \\
\text{CaMKII}_{\text{phospho}} &= (0,0,0,0,1,0,0,0,0,0,0,0,0,0,0,0,0,0,0,1,1,1,1,1,0)\boldsymbol{x},
\end{aligned}$$

and

$$\begin{aligned}
\text{PP2B}_{\text{active}} &= \text{PP2B_CaM_Ca4}, \\
\text{CaMKII}_{\text{active}} &= \text{CaMKII}_{\text{phospho}} + \text{CaMKII_CaM_Ca4},
\end{aligned}$$

then the outputs/read-outs, $\boldsymbol{y}$, that are used correspond to

$$\begin{aligned} y_1 &= \text{Ca}_{\text{bound}}(t^*)/\text{CaM}_{\text{tot}} \\ y_2 &= \text{PP2B\_CaM}(t^*)/(\text{PP2B}(t^*) + \text{PP2B\_CaM}(t^*)) \\ y_3 &= \text{PP2B}_{\text{active}}(t^*)/\text{PP2B}_{\text{tot}} \\ y_4 &= \text{CaMKII}_{\text{phospho}}(t^*)/\text{CaMKII}_{\text{tot}} \\ y_5 &= \int_{t=t'}^{t=t'+30} \text{CaMKII}_{\text{active}}(t) - \text{PP2B}_{\text{active}}(t)dt \end{aligned} \tag{S2}$$

where t^* in our simulations were set to $t^* = 600$, which we assume is near steady state and t' corresponds to the initiation of the Ca-spike train. The output y_5 is normalized by the maximum value from a series of simulations, using different frequencies of the Ca-spike train as input. The normalization is computed by

$$y_{5,i}^{\text{norm}} = y_{5,i}/\max_j(|y_{5,j}|), \tag{S3}$$

where $y_{5,i}$, denotes the output of simulation i.

The different experimental conditions (phenotypes) described in the main text are detailed in Table S2. These correspond to the different input-output curves of Figure 12.3 A-E and Figure 12.4 C in Nair *et al.*, 2014, where each data point i corresponds to a specific input vector $\boldsymbol{u}_i$. The input of one experiment thus corresponds to the matrix $\boldsymbol{U} = (\boldsymbol{u}_1, \ldots, \boldsymbol{u}_n)$, where n is the number of data points, and we denote one experiment with $w = (\boldsymbol{U}, y_{\text{obs}})$, where y_{obs} describes which output function that is observed, e.g. $y_{\text{obs}} = y_3$. Note that for each experimental condition, in fact only one of the input variables is varied and all other kept constant (only one of the rows of $\boldsymbol{U}$ is varied). The experimental data corresponding to one input-output curve are therefore, for ease of notation, denoted by the vector $\boldsymbol{u}_w^{\text{exp}}$ where the components correspond to the values of the varied input variable, and the vector $\boldsymbol{y}_w^{\text{exp}}$, where the components correspond to the values of the observed output variable. The corresponding simulated data are denoted similarly $\boldsymbol{y}_w^{\text{sim}}$ and $\boldsymbol{u}_w^{\text{sim}}$. The subscript w representing a specific phenotype is omitted when it is clear that we only discuss one specific phenotype.

The initial conditions $\boldsymbol{x}_0$ for all simulations were CaM $=$ CaM$_{\text{tot}}$, CaMKII $=$ CaMKII$_{\text{tot}}$, PP2B $=$ PP2B$_{\text{tot}}$ and PP1 $=$ PP1$_{\text{tot}}$, and all other variables set to zero, except for the variable that was varied during the experiment.

Table S1. The different substances in the system presented by Nair et al., 2014.

Number	Name
1	Ca
2	CaM
3	PP2B
4	CaMKII
5	pCaMKII
6	CaM_Ca1
7	CaM_Ca2
8	CaM_Ca3
9	CaM_Ca4
10	PP2B_CaM
11	PP2B_CaM_Ca1
12	PP2B_CaM_Ca2
13	PP2B_CaM_Ca3
14	PP2B _CaM_Ca4
15	CaMKII_CaM
16	CaMKII_CaM_Ca1
17	CaMKII_CaM_Ca2
18	CaMKII_CaM_Ca3
19	CaMKII_CaM_Ca4
20	pCaMKII_CaM
21	pCaMKII_CaM_Ca1
22	pCaMKII_CaM_Ca2
23	pCaMKII_CaM_Ca3
24	pCaMKII_CaM_Ca4
25	PP1

Table S2. Summary of chemical reactions, inputs $\boldsymbol{u}$ and outputs $\boldsymbol{y}$ of the system described in Nair et al., 2014. The different observed phenotypes (denoted ph1-ph6) correspond to different experimental setups and ON means that the corresponding reaction is turned on (exists) in the experiment corresponding to that specific phenotype (subsets of reactions are turned off by setting the corresponding total amount to zero). The prediction (pred) corresponds to Figure 12.4C of Nair et al., 2014. All reactions have reaction rates based on the law of mass action, except the one marked with * where the reaction is more complex (see main text for details). A list of all species can be found in Table S1. The names of the forward reaction kinetic constants k_f are given, the names for k_r and K_d for the reversible reactions follow the same naming convention. The parameter names of the two irreversible reactions are also given.

ID	REACTIONS			ph1	ph2	ph3	ph4	ph5	ph6	pred	k_f (k)
1	CaM + Ca	$\rightleftharpoons$	CaM_Ca1	ON	-	ON	ON	ON	ON	ON	kf*CaM*Ca
2	CaM_Ca1 + Ca	$\rightleftharpoons$	CaM_Ca2	ON	-	ON	ON	ON	ON	ON	kf*CaM_Ca1*Ca
3	CaM_Ca2 + Ca	$\rightleftharpoons$	CaM_Ca3	ON	-	ON	ON	ON	ON	ON	kf*CaM_Ca2*Ca
4	CaM_Ca3 + Ca	$\rightleftharpoons$	CaM_Ca4	ON	-	ON	ON	ON	ON	ON	kf*CaM_Ca3*Ca
5	CaM + PP2B	$\rightleftharpoons$	PP2B_CaM	-	ON	ON	ON	-	-	ON	kf*CaM*PP2B
6	CaM_Ca1 + PP2B	$\rightleftharpoons$	PP2B_CaM_Ca1	-	-	ON	ON	-	-	ON	kf*CaM_Ca1*PP2B
7	CaM_Ca2 + PP2B	$\rightleftharpoons$	PP2B_CaM_Ca2	-	-	ON	ON	-	-	ON	kf*CaM_Ca2*PP2B
8	CaM_Ca3 + PP2B	$\rightleftharpoons$	PP2B_CaM_Ca3	-	-	ON	ON	-	-	ON	kf*CaM_Ca3*PP2B
9	CaM_Ca4 + PP2B	$\rightleftharpoons$	PP2B_CaM_Ca4	-	-	ON	ON	-	-	ON	kf*CaM_Ca4*PP2B
10	PP2B_CaM + Ca	$\rightleftharpoons$	PP2B_CaM_Ca1	-	-	ON	ON	-	-	ON	kf*PP2B_CaM*Ca
11	PP2B_CaM_Ca1 + Ca	$\rightleftharpoons$	PP2B_CaM_Ca2	-	-	ON	ON	-	-	ON	kf*PP2B_CaM_Ca1*Ca
12	PP2B_CaM_Ca2 + Ca	$\rightleftharpoons$	PP2B_CaM_Ca3	-	-	ON	ON	-	-	ON	kf*PP2B_CaM_Ca2*Ca
13	PP2B_CaM_Ca3 + Ca	$\rightleftharpoons$	PP2B_CaM_Ca4	-	-	ON	ON	-	-	ON	kf*PP2B_CaM_Ca3*Ca
14	CaM + CaMKII	$\rightleftharpoons$	CaMKII_CaM	-	-	-	-	ON	ON	ON	kf*CaM*CaMKII
15	CaM_Ca1 + CaMKII	$\rightleftharpoons$	CaMKII_CaM_Ca1	-	-	-	-	ON	ON	ON	kf*CaM_Ca1*CaMKII
16	CaM_Ca2 + CaMKII	$\rightleftharpoons$	CaMKII_CaM_Ca2	-	-	-	-	ON	ON	ON	kf*CaM_Ca2*CaMKII
17	CaM_Ca3 + CaMKII	$\rightleftharpoons$	CaMKII_CaM_Ca3	-	-	-	-	ON	ON	ON	kf*CaM_Ca3*CaMKII
18	CaM_Ca4 + CaMKII	$\rightleftharpoons$	CaMKII_CaM_Ca4	-	-	-	-	ON	ON	ON	kf*CaM_Ca4*CaMKII
19	CaMKII_CaM + Ca	$\rightleftharpoons$	CaMKII_CaM_Ca1	-	-	-	-	ON	ON	ON	kf*CaMKII_CaM*Ca
20	CaMKII_CaM_Ca1 + Ca	$\rightleftharpoons$	CaMKII_CaM_Ca2	-	-	-	-	ON	ON	ON	kf*CaMKII_CaM_Ca1*Ca
21	CaMKII_CaM_Ca2 + Ca	$\rightleftharpoons$	CaMKII_CaM_Ca3	-	-	-	-	ON	ON	ON	kf*CaMKII_CaM_Ca2*Ca
22	CaMKII_CaM_Ca3 + Ca	$\rightleftharpoons$	CaMKII_CaM_Ca4	-	-	-	-	ON	ON	ON	kf*CaMKII_CaM_Ca3*Ca
23	CaM_Ca4 + pCaMKII	$\rightleftharpoons$	pCaMKII_CaM_Ca4	-	-	-	-	ON	ON	ON	kf*CaM_Ca4*pCaMKII
24	CaM_Ca3 + pCaMKII	$\rightleftharpoons$	pCaMKII_CaM_Ca3	-	-	-	-	ON	ON	ON	kf*CaM_Ca3*pCaMKII
25	CaM_Ca2 + pCaMKII	$\rightleftharpoons$	pCaMKII_CaM_Ca2	-	-	-	-	ON	ON	ON	kf*CaM_Ca2*pCaMKII
26	CaM_Ca1 + pCaMKII	$\rightleftharpoons$	pCaMKII_CaM_Ca1	-	-	-	-	ON	ON	ON	kf*CaM_Ca1*pCaMKII
27	CaM + pCaMKII	$\rightleftharpoons$	pCaMKII_CaM	-	-	-	-	ON	ON	ON	kf*CaM*pCaMKII
28	pCaMKII_CaM + Ca	$\rightleftharpoons$	pCaMKII_CaM_Ca1	-	-	-	-	ON	ON	ON	kf*pCaMKII_CaM*Ca
29	pCaMKII_CaM_Ca1 + Ca	$\rightleftharpoons$	pCaMKII_CaM_Ca2	-	-	-	-	ON	ON	ON	kf*pCaMKII_CaM_Ca1*Ca
30	pCaMKII_CaM_Ca2 + Ca	$\rightleftharpoons$	pCaMKII_CaM_Ca3	-	-	-	-	ON	ON	ON	kf*pCaMKII_CaM_Ca2*Ca
31	pCaMKII_CaM_Ca3 + Ca	$\rightleftharpoons$	pCaMKII_CaM_Ca4	-	-	-	-	ON	ON	ON	kf*pCaMKII_CaM_Ca3*Ca
32	CaMKII_CaM_Ca4	$\rightharpoonup^\star$	pCaMKII_CaM_Ca4	-	-	-	-	ON	ON	ON	kautMax*
33	pCaMKII + PP1	$\rightleftharpoons$	PP1_pCaMKII	-	-	-	-	-	-	ON	kf*pCaMKII*PP1
34	PP1_pCaMKII	$\rightharpoonup$	CaMKII + PP1	-	-	-	-	-	-	ON	k*PP1_pCaMKII
	**INPUT ($\boldsymbol{u}$)**										
1	[Ca](t)			varied**	-	varied**	varied**	varied**	varied**	varied***	
1	CaM_{tot}			25000nM	varied**	30nM	300nM	5000nM	2000nM	10000nM	
2	PP2B_{tot}			-	100 nM	3 nM	3nM	-	-	4000nM	
3	$\text{CaMKII}_{\text{tot}}$			-	-	-	-	5000nM	200nM	20000nM	
4	PP1_{tot}			-	-	-	-	-	-	5000nM	
	**OUTPUT ($\boldsymbol{y}$)**										
	Observable/Readout			y_1	y_2	y_3 (in %)	y_3 (in %)	y_1	y_4 (in %)	y_5^{norm}	Defined in Equations S2 and S3.
	Type			st. st.	st. st.	st. st.	st. st.	$\approx$ st. st.	$\approx$ st. st.	after spike train	

* The reaction rate of reaction 32 is modeled by a function described in S1.2. ** The input variable has a time constant value that is varied for each new simulation/experiment. *** The input variable corresponds to a time dependent function, a Ca-spike train with a new frequency for each simulation. Abbreviations: st. st. = steady state

Table S3. The thermodynamic constraint rules connecting different equilibrium constants of the model due to multiple possible reaction paths when one species is being converted into another.

[K_d*CaM_Ca3*PP2B]	=	([K_d*CaM_Ca3*Ca] * [K_d*CaM_Ca4*PP2B]) / [K_d*PP2B_CaM_Ca3*Ca]
[K_d*CaM_Ca2*PP2B]	=	([K_d*CaM_Ca2*Ca] * [K_d*CaM_Ca3*PP2B]) / [K_d*PP2B_CaM_Ca2*Ca]
[K_d*CaM_Ca1*PP2B]	=	([K_d*CaM_Ca1*Ca] * [K_d*CaM_Ca2*PP2B]) / [K_d*PP2B_CaM_Ca1*Ca]
[K_d*CaM*PP2B]	=	([K_d*CaM*Ca] * [K_d*CaM_Ca1*PP2B]) / [K_d*PP2B_CaM*Ca]
[K_d*CaM_Ca3*CaMKII]	=	([K_d*CaM_Ca3*Ca] * [K_d*CaM_Ca4*CaMKII]) / [K_d*CaMKII_CaM_Ca3*Ca]
[K_d*CaM_Ca2*CaMKII]	=	([K_d*CaM_Ca2*Ca] * [K_d*CaM_Ca3*CaMKII]) / [K_d*CaMKII_CaM_Ca2*Ca]
[K_d*CaM_Ca1*CaMKII]	=	([K_d*CaM_Ca1*Ca] * [K_d*CaM_Ca2*CaMKII]) / [K_d*CaMKII_CaM_Ca1*Ca]
[K_d*CaM*CaMKII]	=	([K_d*CaM*Ca] * [K_d*CaM_Ca1*CaMKII]) / [K_d*CaMKII_CaM*Ca]
[K_d*CaM_Ca3*pCaMKII]	=	([K_d*CaM_Ca3*Ca] * [K_d*CaM_Ca4*pCaMKII]) / [K_d*pCaMKII_CaM_Ca3*Ca]
[K_d*CaM_Ca2*pCaMKII]	=	([K_d*CaM_Ca2*Ca] * [K_d*CaM_Ca3*pCaMKII]) / [K_d*pCaMKII_CaM_Ca2*Ca]
[K_d*CaM_Ca1*pCaMKII]	=	([K_d*CaM_Ca1*Ca] * [K_d*CaM_Ca2*pCaMKII]) / [K_d*pCaMKII_CaM_Ca1*Ca]
[K_d*CaM*pCaMKII]	=	([K_d*CaM*Ca] * [K_d*CaM_Ca1*pCaMKII]) / [K_d*pCaMKII_CaM*Ca]

S2 Details of the ABC-MCMC uncertainty quantification

S2.1 A short introduction to copulas

Lets assume that our aim is to describe the multivariate distribution for the random vector $\boldsymbol{X} = (X_1, \ldots, X_d)'$ in some way. The elements of $\boldsymbol{X}$ are assumed to have continuous marginal distribution functions; $F_i(x_i) = P(X_i \leq x_i)$. Copulas are multivariate probability distributions which describe the dependence structure between the stochastic variables. The copula is a function that connects the multivariate distribution function to the marginal ones (F_i) as follows.

$$F(\boldsymbol{x}) = C(F_1(x_1), \ldots, F_d(x_d))$$

It can be shown (Sklar's theorem) that for continuous marginal distributions, C is unique. The elements of the vector $(U_1, \ldots, U_d) = (F_1(x_1), \ldots, F_d(x_d))$ are by definition uniformly distributed. Hence copulas can be viewed as multivariate distribution functions whose one-dimensional margins are uniform on the interval $[0, 1]$ (Nelsen, 2006).

The pair-copula decomposition of a multivariate distribution is useful in order to describe the distribution in question. Consider the vector $\boldsymbol{X}$ and the corresponding density function $f(\boldsymbol{x}) = f(x_1, \ldots, x_d)$ which can be factorized as

$$f(x_1, \ldots, x_d) = f_d(x_d) \cdot f(x_{d-1}|x_d) \cdot f(x_{d-2}|x_{d-1}, x_d) \cdot \ldots \cdot f(x_1|x_2, \ldots, x_d)$$

It can be shown that the multivariate (joint) density can be represented by a number of appropriate pair-copulas times the conditional marginal densities based on this factorization. For a vector with three components, we have for example (by use of the chain rule)

$$f(x_1, x_2, x_3) = f_1(x_1) f(x_2|x_1) f(x_3|x_1, x_2)$$

and

$$f(x_2|x_1) = \frac{f(x_1, x_2)}{f_1(x_1)} = \frac{c_{1,2}(F_1(x_1), F_2(x_2)) f_1(x_1) f_2(x_2)}{f_1(x_1)} = c_{1,2}(F_1(x_1), F_2(x_2)) f_2(x_2)$$

$$f(x_3|x_1, x_2) = \ldots = c_{2,3|1}(F(x_2|x_1), F(x_3|x_1)) c_{1,3}(F_1(x_1), F_3(x_3)) f_3(x_3)$$

This gives

$$f(x_1, x_2, x_3) = c_{1,2}(F_1(x_1), F_2(x_2)) c_{2,3|1}(F(x_2|x_1), F(x_3|x_1)) c_{1,3}(F_1(x_1), F_3(x_3)) f_1(x_1) f_3(x_3) f_2(x_2)$$

The copula pairs can be chosen independently of each other giving a wide range of possible dependence structures, especially for high-dimensional distributions. Graphical models called vines were introduced to arrange the pair copulas in a tree structure (see e.g Bedford and Cooke, 2002 and Aas *et al.*, 2009). C- and D-vines are constructed by choosing a specific order of the variables included. R-vines in turn are a more flexible superclass of C- and D-vines.

S2.2 Example of using copulas

Let's assume we work with a simple bivariate distribution and that we have a bivariate sample $\boldsymbol{x} = [\boldsymbol{x}_1, \boldsymbol{x}_2]$. For each variable X_1 and X_2, we estimate a cumulative distribution $P(X_1 \leq x_1)$ and $P(X_2 \leq x_2)$ (this can be done in R using e.g. the kcde or ecdf functions and in MATLAB using the ksdensity function). We evaluate these cumulative densities in the sample points $[\boldsymbol{x}_1, \boldsymbol{x}_2]$ and denote this information $\boldsymbol{z} = [\boldsymbol{z}_1, \boldsymbol{z}_2]$. This step maps the sample points ($\boldsymbol{x}$) into the $[0, 1]$ space and we fit a copula to $\boldsymbol{z}$ in this space.

We take a single sample $\boldsymbol{s}$ from the copula. $\boldsymbol{s}$ will have two values which are observations in $[0, 1]$. To translate this information back into the original sample space, we can use the inverse cumulative density, or simply interpolate from the $[0, 1]$ space back to the original space for each variable using $\boldsymbol{z}_1$ and $\boldsymbol{x}_1$ and $\boldsymbol{z}_2$ and $\boldsymbol{x}_2$, respectively.

If we take multiple sample points from the copula and transform them back to the original space, we will get a sample on the original scale with the estimated marginal densities under the dependency modelled by the copula.

We illustrate what results can look like if taking this approach in Figure S1 where we have modelled two variables exhibiting a curvature in sample space with a copula.

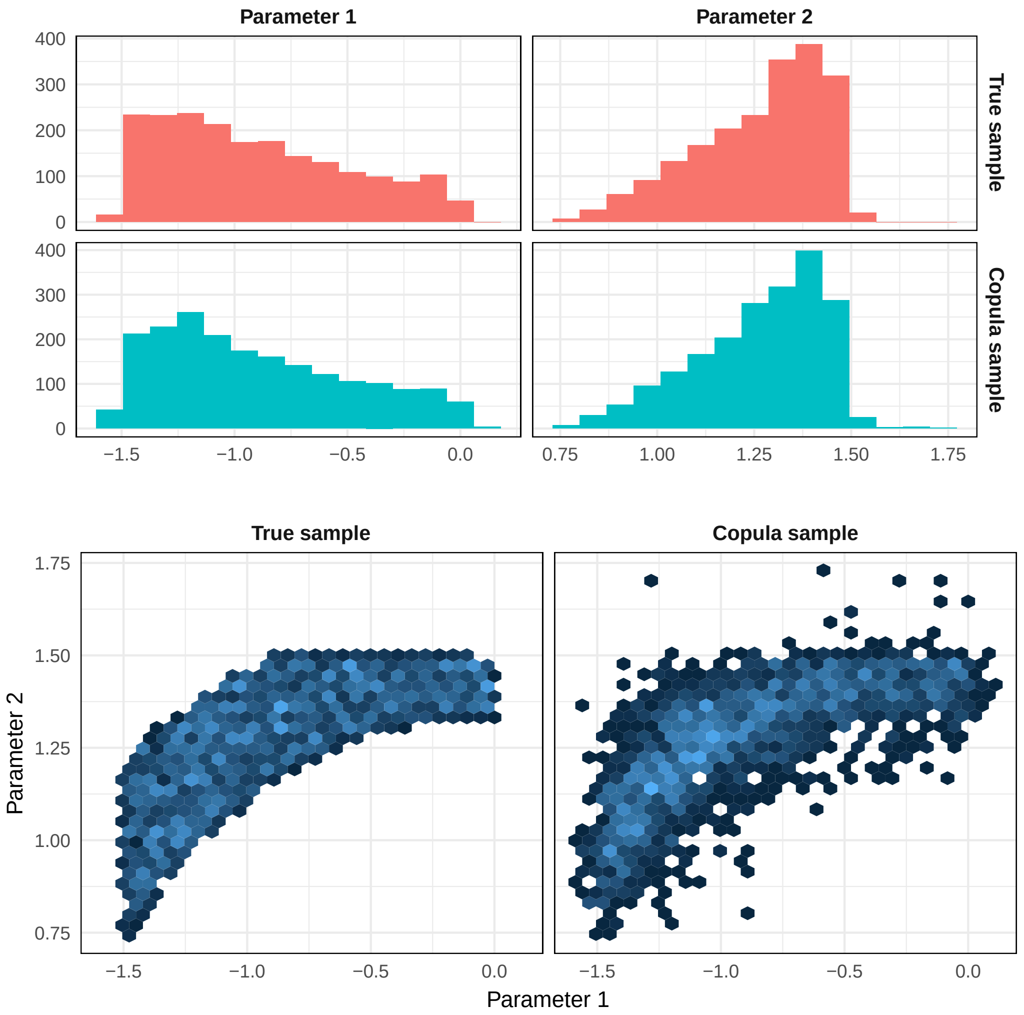

Fig. S1. Example of modelling two dependent variables with a copula. The top panel shows the histograms for the true sample and the copula sample for each of the two parameters. The bottom panel shows the two-dimensional densities of the two samples. 2000 sampled points are used in each sample.

S2.3 Normalization of experimental and simulated data for the uncertainty quantification

Let the vector $\boldsymbol{u}_w^{\text{sim}}$ denote the (varied) input of one phenotype and the vector $\boldsymbol{y}_w^{\text{sim}}$ the corresponding output (for simplicity we leave out the phenotype subscript w below). In order to be able to compare different experimental setups, we log-transformed the input u_j^{sim} and set $z_j^{\text{sim}} = \log(u_j^{\text{sim}})$ where j indexes the different components of the input vector. The log-transformed input z_j^{sim} and the output y_j^{sim} in each simulation were normalized according to

$$z_{N,j}^{\text{sim}} = \frac{z_j^{\text{sim}} - \min(\boldsymbol{z}^{\text{sim}})}{\max(\boldsymbol{z}^{\text{sim}}) - \min(\boldsymbol{z}^{\text{sim}})}, \; y_{N,j}^{\text{sim}} = \frac{y_j^{\text{sim}} - \min(\boldsymbol{y}^{\text{sim}})}{\max(\boldsymbol{y}^{\text{sim}}) - \min(\boldsymbol{y}^{\text{sim}})} \tag{S4}$$

where $z_{N,j}^{\text{sim}}$ is the normalized version of the jth component z_j^{sim}, and similarly for $y_{N,j}^{\text{sim}}$. The experimental data $\boldsymbol{y}^{\text{exp}}$ and the experimental input (on the log-scale) $\boldsymbol{z}^{\text{exp}}$ were normalized in the same way (using min/max values from the simulated input and outputs).

S2.4 Distance measure used in the ABC sampling

We have used the following distance measure:

$$\rho = \max_i \left\{ \min_j \left\{ \sqrt{\left(\frac{z^{\text{sim}}_{N,j} - z^{\text{exp}}_{N,i}}{0.5}\right)^2 + \left(\frac{y^{\text{sim}}_{N,j} - y^{\text{exp}}_{N,i}}{0.5}\right)^2} \right\} \right\}$$

where $(z^{\text{exp}}_{N,i}, y^{\text{exp}}_{N,i})$ is the normalized experimental data point i, $i = 1 \ldots n$, and $(z^{\text{sim}}_{N,j}, y^{\text{sim}}_{N,j})$ is the normalized simulated data point j, $j = 1 \ldots m$, and $m >> n$. The simulated data points were retrieved using a grid on the x-axis (in the order of 100 points). Checking whether $\rho < \delta$, where δ is the chosen ABC-threshold, corresponds to defining a circle around each normalized experimental point and checking that all circles have a part of the simulated curve passing through. The circles have a radius equal to 0.5δ, which is a deviation of $100\delta\%$ of the average normalized output (0.5) for all points on the curve, The value of δ was set to 0.1, corresponding to a 10% deviation. We adopt this scheme in order to account for noise in both input and output variables.

S2.5 Pseudocode for ABC-MCMC sampling with copulas

For each data set corresponding to phenotypes 1-6 the following steps are performed (with the exception of step P3 for phenotype 6):

P1 **Pre-calibration sampling** (In order to construct a covariance matrix for the MCMC transition kernel as well as finding a starting point for the MCMC chain.)
P2 **ABC-MCMC sampling of viable space** (First an adaptive threshold is used to quickly reach the viable space, and next the viable space is sampled.)
P3 **Copula estimation** (A copula is estimated based on a sample from the posterior distribution of the data set in question.)

For the first dataset (phenotype 1) a uniform marginal prior is used for the parameters, for datasets 2-6 the copula from the previous data set is used as a prior. The details of each step is described below, where $\boldsymbol{\theta}$ is a vector of size p (the number of model parameters), the vector $\boldsymbol{y}^{\text{sim}}$ denotes the simulated output of one phenotype, and the vector $\boldsymbol{y}^{\text{exp}}$ denotes the corresponding experimental output.

P1 **Pre-calibration sampling**
- P1.1 Sample independently from the prior $f(\boldsymbol{\theta})$, n number of times to get a set of samples $\boldsymbol{P} = (\boldsymbol{\theta}_1, \ldots, \boldsymbol{\theta}_n)$.
- P1.2 Generate simulated output $\boldsymbol{S} = (\boldsymbol{y}^{\text{sim}}_1, \ldots, \boldsymbol{y}^{\text{sim}}_n)$ from the model for all samples in $\boldsymbol{P}$.
- P1.3 Compute distances $\boldsymbol{D} = (\rho_1, \ldots, \rho_n)$ to the experimental data $\boldsymbol{y}^{\text{exp}}$ by the distance measure $\rho(\boldsymbol{y}^{\text{sim}}_i, \boldsymbol{y}^{\text{exp}})$ for all $\boldsymbol{y}^{\text{sim}}_i$ in $\boldsymbol{S}$.
- P1.4 Sort $\boldsymbol{P}$ according to distances in $\boldsymbol{D}$. Save the subsample $\boldsymbol{P}'$ corresponding to the 0.01 fraction of smallest distances (in rare cases $> 0.01 \cdot n$ samples meet $\rho \leq \delta$, where δ is the chosen similarity cutoff, then all these are kept.)
- P1.5 Estimate a covariance matrix $\boldsymbol{\Sigma}_1$ from $\boldsymbol{P}'$ and scale it appropriately in order to use it in transition kernel Q. Also define a diagonal matrix $\boldsymbol{\Sigma}_0 = diag(v_1, \ldots, v_p)$ of size p (we choose $\boldsymbol{\Sigma}_0$ to be a diagonal version of $\boldsymbol{\Sigma}_1$ with smaller variances v_i). Pick a random starting point $\boldsymbol{\theta}'$ from $\boldsymbol{P}'$.

P2 **ABC-MCMC sampling from viable space**
- P2.1 Generate $\boldsymbol{y}^{\text{sim}'}$ using the model with parameters $\boldsymbol{\theta}'$.
- P2.2 Set the distance $\delta_{cur} = \rho(\boldsymbol{y}^{\text{sim}'}, \boldsymbol{y}^{\text{exp}})$ (*current* value of the adaptive threshold).
- P2.3 Propose a move from current $\boldsymbol{\theta}'$ to $\boldsymbol{\theta}''$ according to the transition kernel $Q(\boldsymbol{\theta}' \to \boldsymbol{\theta}'')$. The proposed point $\boldsymbol{\theta}''$ is drawn from distribution F where $F = 0.95F_1 + 0.05F_0$ in which $F_1 \sim MVN(\boldsymbol{\theta}', \boldsymbol{\Sigma}_1)$ and $F_0 \sim MVN(\boldsymbol{\theta}', \boldsymbol{\Sigma}_0)$.
- P2.4 Generate $\boldsymbol{y}^{\text{sim}''}$ using the model with parameters $\boldsymbol{\theta}''$ and calculate the distance $\delta_{can} = \rho(\boldsymbol{y}^{\text{sim}''}, \boldsymbol{y}^{\text{exp}})$ (*candidate* threshold).
- P2.5 If $\delta_{can} \leq \max(\delta, \delta_{cur})$ go to P2.6 else return to P2.3.
- P2.6 Calculate $h(\boldsymbol{\theta}', \boldsymbol{\theta}'') = \min(1, f(\boldsymbol{\theta}'')/f(\boldsymbol{\theta}'))$ where f is the prior (copula) distribution.
- P2.7 With probability h, set $\boldsymbol{\theta}' = \boldsymbol{\theta}''$ and $\delta_{cur} = \delta_{can}$.
- P2.8 If $\delta_{cur} \leq \delta$ and $\boldsymbol{\theta}' = \boldsymbol{\theta}''$, accept $\boldsymbol{\theta}'$ as a sample point. Go to P2.3.

P3 **Copula estimation**
- P3.1 Filter the resulting ABC-MCMC samples and keep only sample points that fit all tested phenotypes (e.g. for phenotype 2, only parameters that fit both phenotype 1 and 2 would be kept).
- P3.2 Select a random subset of size approximately 5000 samples (edges of sampled space are explicitly added as well) $(\boldsymbol{\theta}_1, \ldots \boldsymbol{\theta}_p)$ from the set defined in P3.1.
- P3.3 Estimate the marginal cumulative distribution for each parameter k by using the vector $\boldsymbol{\theta}_k$ and evaluate the cumulative distribution in the sample points $\boldsymbol{\theta}_k$ and denote this $\boldsymbol{z}_k$. Set $\boldsymbol{z} = (\boldsymbol{z}_1, \ldots \boldsymbol{z}_p)$.
- P3.4 Fit an R-vine copula to $\boldsymbol{z}$.

Some notes:
- In the ABC-MCMC step, we use a mixture distribution to propose new steps, in order to build in some flexibility into the transition kernel.
- If the MCMC chain gets stuck for more than 500 iterations in one sample point, the chain is terminated. This happened a few times in our model fitting, and was most often due to the fact that the chain got stuck before reaching the viable space.
- Several MCMC chains are run in the ABC-MCMC step, with different random starting points but the same transition kernel, and all samples are merged before the next step.
- A subset of size approximately 5000 samples from the ABC-MCMC step is used to fit the copula. The size of this subset was chosen in order to maintain a good copula fit while speeding up the fitting process.

S2.6 Validation of the ABC-MCMC-copula approach

The ABC-MCMC-copula approach was validated in two different ways. First through a small test case for which it is possible to compare the posterior distributions with the corresponding ones obtained from pure rejection sampling (which we consider to be as close to the ground truth as possible), and secondly by analysing the distributions obtained in each step of the inverse quantification (i.e. after fitting to each phenotype) and comparing that to the corresponding approximate distributions from the copulas.

The smaller test case consisted of the submodel corresponding to phenotypes 1-3 of Table S2. For this smaller model we can use the analytical steady state solutions (S8), (S11) and (S17) to compare with steady state data, which makes it possible to use pure rejection sampling to sample the posterior distribution. In phenotypes 1-3, only a subset of the parameters of the full model are included, but the dependencies between some of these parameters are strong.

The pure rejection sampling was done from the uniform priors and in sequence, starting with phenotype 1. It should be noted that performing rejection sampling with the chosen priors needed the evaluation of more than 300 million sampling points in order to find 5000 points that fitted all three phenotypes. We compare a sample from the pure rejection sampling approach (red in Figure S2) to a sample from the sequential ABC-MCMC-copula approach (blue in Figure S2), as well as an approach using only copula fitting together with rejection sampling (green in Figure S2). The third approach was included to evaluate the effect of the copulas alone, without the MCMC part. All three methods use the same ABC distance criterion to accept sample points (as described in Section S2.4).

Both the ABC-MCMC-copula and rejection-copula approach have marginal distributions which are very similar to the pure rejection approach. It can be noted that there is a small tail of the distribution, visible for the parameters K_d*CaM_Ca1*Ca and K_d*CaM_Ca2*Ca, which are not reproduced by the ABC-MCMC-copula method and just barely for the copula rejection approach. Apart from this tail, which makes up a small fraction of the sample, we consider the ABC-MCMC-copula approach to reproduce the true posterior very well for this small test. In Figure S3 we also show density plots of the two most correlated parameter pairs for all three methods. The overall dependencies between the variables are captured well with the ABC-MCMC copula approach.

The second validation evaluated how well the copula method models the posterior distribution at each step of the full sequential ABC-MCMC-copula approach. For each step, the MCMC sample from the posterior distribution was compared to a sample from a copula fitted to the same distribution. The comparison consisted of examining correlation plots of all parameters as well as two-dimensional density plots of the parameter pairs with the lowest and highest Kullback-Leibler divergence (KLD) between the MCMC and copula samples. The linear correlations are similar/almost identical between the ABC-MCMC sample and the copula sample (Figure S4) and even if we look at the parameter pair with the highest KLD the two samples have a similar distribution (Figure S5).

Ideally one would like to have a goodness of fit score for each copula fitting, and there are versions of such tests available. However, current implementations of these tests are limited to a subset of copula families, and hence not applicable to our setting. Until the implementations of these tests have been further developed, we recommend visual inspection together with a KLD approach as described above to evaluate the adequacy of the copula.

S2.7 Implementation of copulas and usage in ABC-MCMC framework

Copulas are implemented in several software suites. We have tested and evaluated copulas in both R and MATLAB. Some copulas are implemented in MATLAB in the Statistics and Machine Learning Toolbox. A set of bivariate Archimedean copulas are available, but multivariate copulas with more than 2 variables are limited to Gaussian and t-copulas. An external toolbox is available called VineCopulaMatlab toolbox (https://github.com/MalteKurz/VineCopulaMatlab), which has a rich set of functions to work with C-and D-vine copulas. In R, there are several packages with different capabilities that implement copulas. For vine copulas, there is the CDVine package (Brechmann and Schepsmeier, 2013) and a development of this package called the VineCopula package (Schepsmeier *et al.*, 2018) which implements a superclass of C- and D-vine copulas called regular vines (R-vines).

When initiating the evaluation of copulas to use in this framework, we started by using Gaussian copulas which often are easy to fit and simple to handle. Unfortunately, such copulas did not adequately fit the sampled distributions (evaluated via comparing samples from the copula with the observed samples). We then turned to vine copulas and tested first the VineCopulaMatlab toolbox which implements C- and D-vine copulas. However, due to limitations with portability for the Matlab toolbox, we also implemented our ABC-MCMC methodology with copulas in R based on capabilities in the VineCopula package. The function RVineStructureSelect was used to select the structure of the vine copula and fit parameters of the chosen bivariate copulas. Both in MATLAB and R, there are more than 40 copula families that are chosen from when selecting the pair copulas, e.g. Gaussian, Clayton, Gumbel, Frank, and Joe copulas and their rotations.

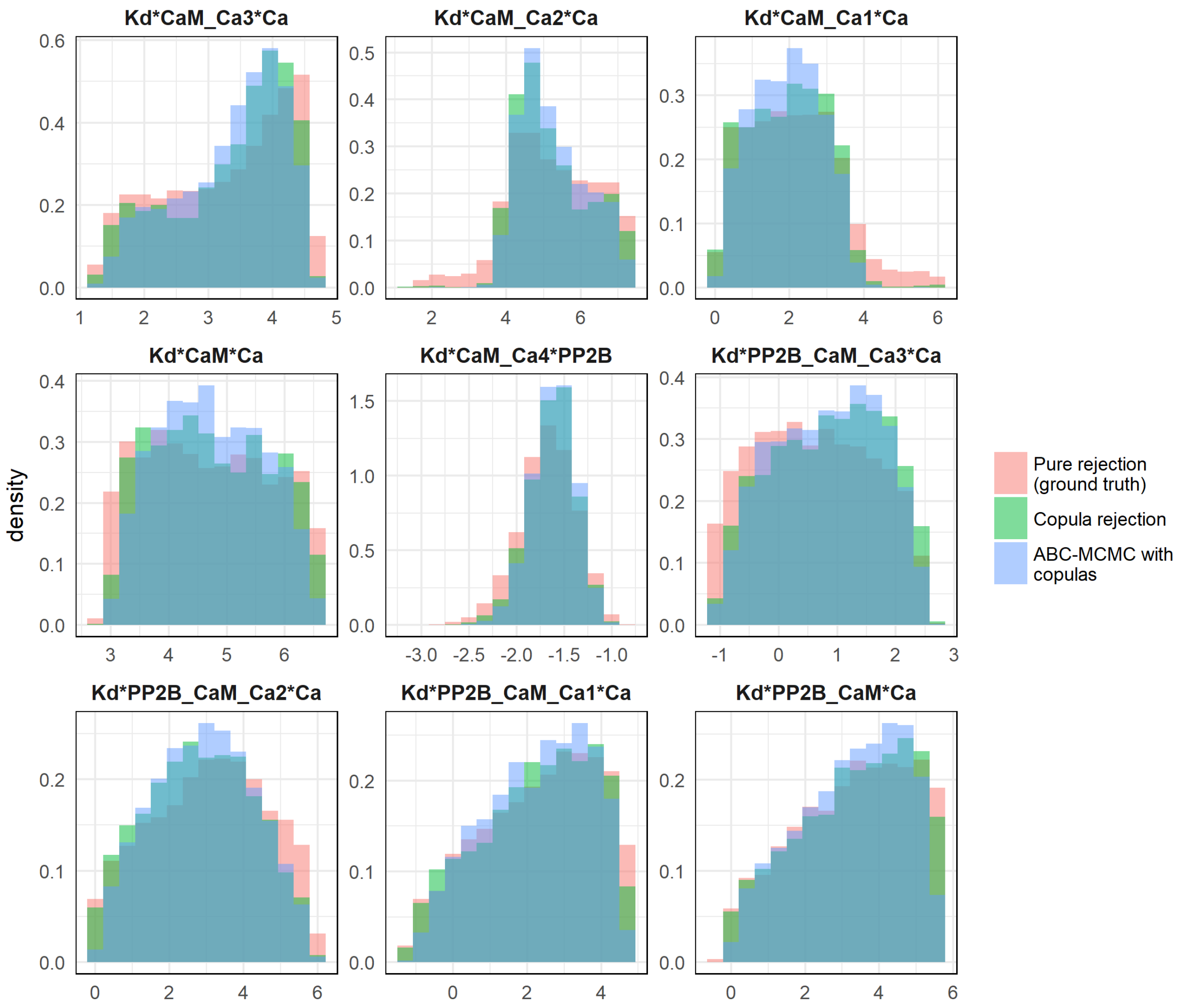

Fig. S2. Overlaid histograms of samples for the nine parameters relevant in a submodel which has been fitted with different methods to phenotypes 1-3.

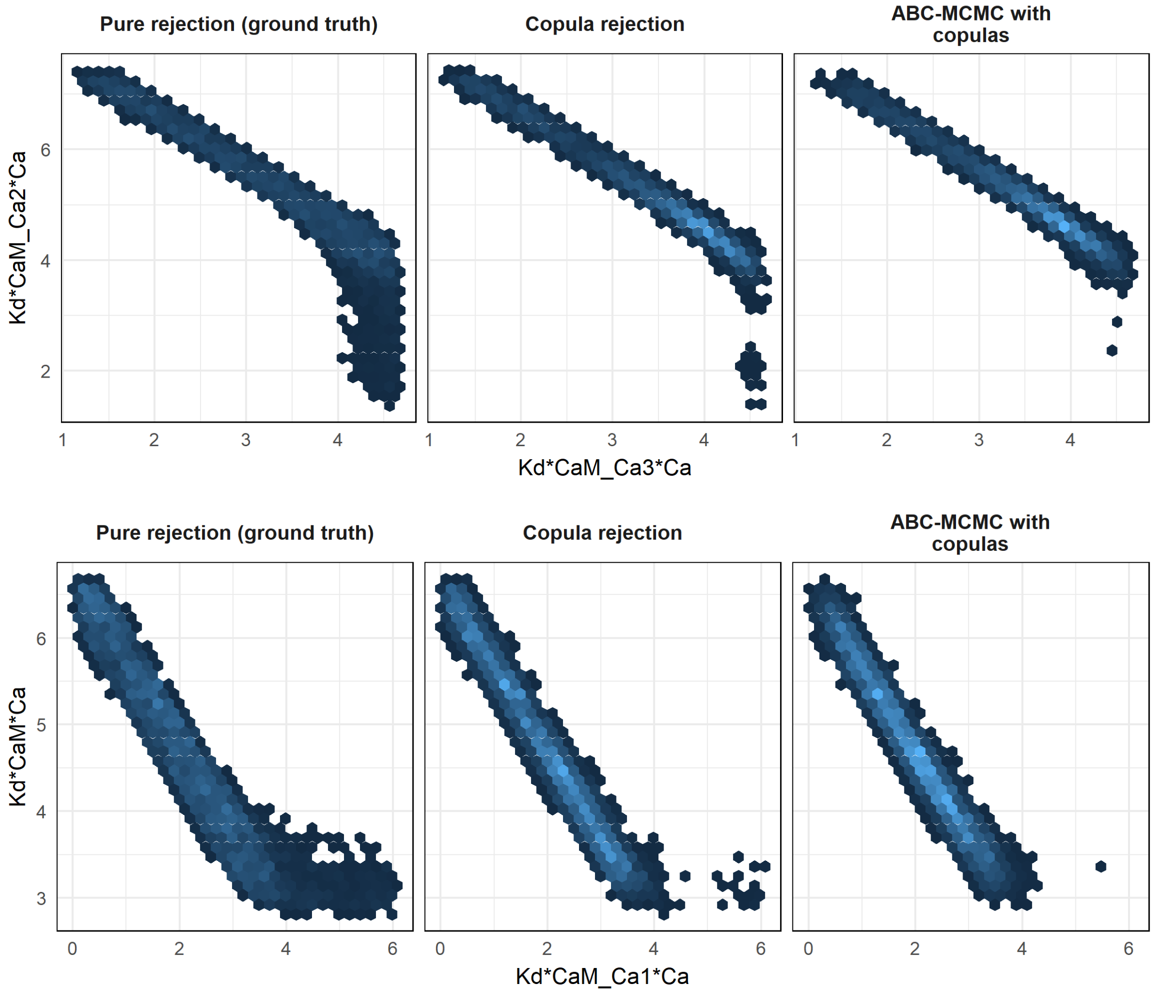

Fig. S3. Two-dimensional density plots of the samples for the two most correlated parameter pairs fitted with different methods to phenotypes 1-3.

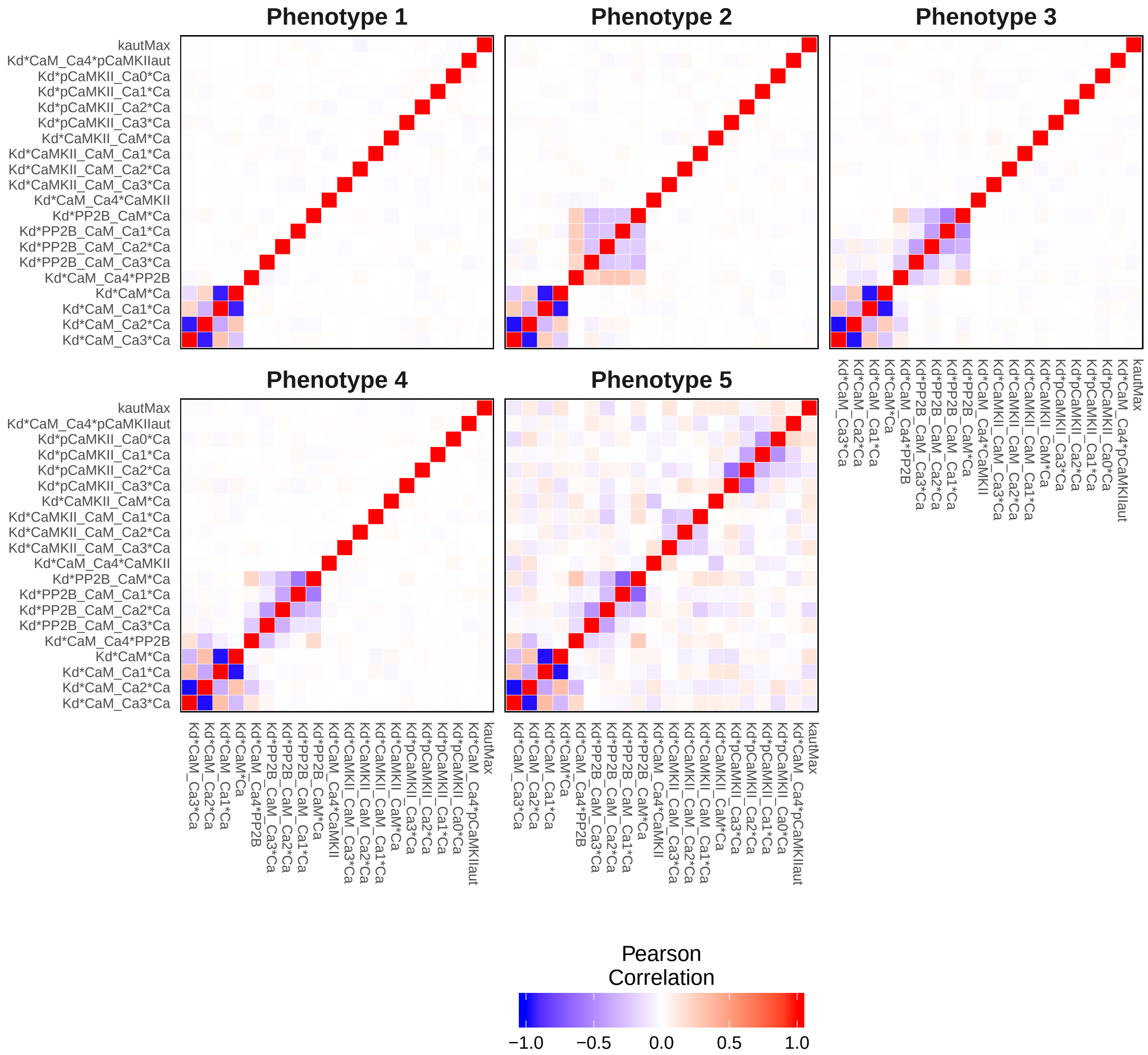

Fig. S4. Correlation plots over all parameters based on the sample from ABC-MCMC and a sample from the corresponding copula for each fitted phenotype (which are done in sequence). The bottom triangular part of the matrices correspond to the copula sample and the top part to the MCMC sample.

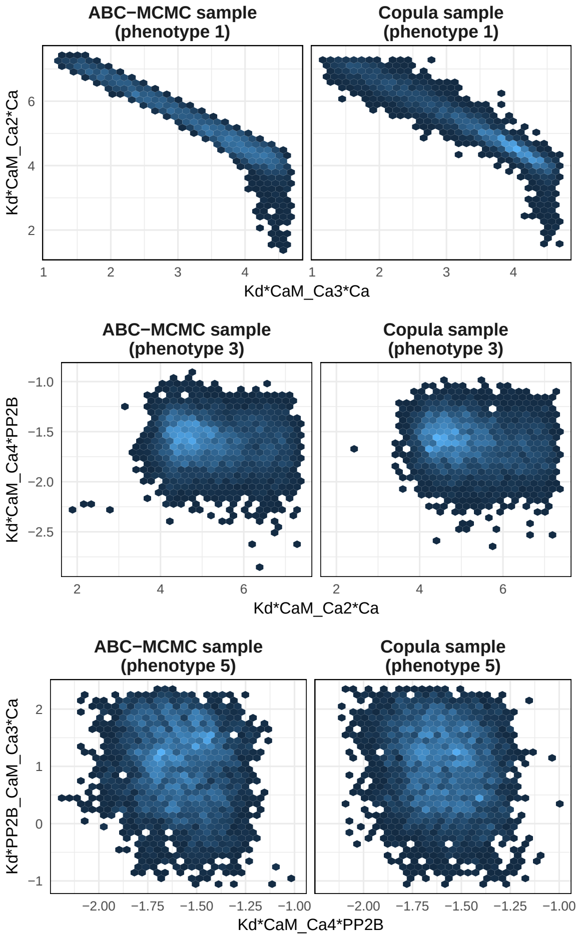

Fig. S5. Two-dimensional density plots over the parameter pairs with the highest KLD between the MCMC and copula samples for each fitted phenotype.

S3 Global sensitivity analysis

S3.1 Normalisation and scale

The sensitivity analysis were performed on the output corresponding to the prediction, $\boldsymbol{y}^{\text{pred}}$. This is a vector which normalized components corresponds to $y_{5,i}^{\text{norm}}$, defined in Equation S3. The binning of the parameters in the sensitivity analysis was performed on the log10-scale.

S3.2 Sensitivity indices

We here describe the calculation of the sensitivity index S_i corresponding to the parameter Θ_i and the predicted output Y, over the posterior distribution retrieved from the uncertainty quantification (Y and Θ_i are stochastic variables, and Y corresponds to one component of the vector $\boldsymbol{y}^{\text{pred}}$). The sensitivity index is defined by $S_i = V_{\Theta_i}(E_{\boldsymbol{\Theta}_{-i}}(Y|\Theta_i))/V(Y)$, where $\boldsymbol{\Theta}_{-i}$ corresponds to all elements of $\boldsymbol{\Theta}$ except Θ_i.

This calculation was inspired by Saltelli *et al.*, 2004 (chapter 5.10), but with major modifications in order to use the existing posterior sample produced from the ABC. The prediction y is a (complicated) function of the parameters $y = h(\boldsymbol{\theta})$), i.e. each sample point $\boldsymbol{\theta}$ has a corresponding $\boldsymbol{y}$ value (y also depends on the input $\boldsymbol{u}$, but to simplify the argument we here assume a specific input, $\boldsymbol{u} = \boldsymbol{u}^*$). We first want to calculate the inner conditional expected value $E_{\boldsymbol{\Theta}_{-i}}(Y|\Theta_i = \theta_i^*)$, in principle for all different values of $\theta_i^* \in \Theta_i$. We approximate this by binning the posterior sample with respect to Θ_i, into m number of bins of size σ, with midpoint $\theta_{i,j}^*$, and indexed by $j = 1, \ldots, m$, i.e. $E_{\boldsymbol{\Theta}_{-i}}(Y|\Theta_i = \theta_i^*) \approx E(Y|\Theta_i \in \delta\theta_{i,j}) = E(Y)_{i,j}$, where $\delta\theta_{i,j} = \{\theta_i | \theta_{i,j}^* - \frac{1}{2}\sigma < \theta_i < \theta_{i,j}^* + \frac{1}{2}\sigma\}$. Let $p_j(y|\theta_i \in \delta\theta_{i,j})$ be the conditional distribution of Y given that Θ_i is in the j:th bin. We denote the corresponding subsample from the posterior distribution $\boldsymbol{y}_{i,j} = (y_{i,j}^1, y_{i,j}^2, \ldots, y_{i,j}^{r_j})$, where r_j is the total number of points in the j:th bin. The sample conditional and unconditional means are:

$$
\begin{aligned}
E(Y)_{ij} &= \bar{y}_{i,j} = \frac{1}{r_j}\sum_{k=1}^{r_j} y_{i,j}^k \\
E(Y) &= \bar{y} = \frac{1}{\sum_{j=1}^m r_j}\sum_{j=1}^{m}\sum_{k=1}^{r_j} y_{i,j}^k
\end{aligned}
$$

The sensitivity index is calculated by:

$$
\begin{aligned}
S_i &= \frac{V_i}{V} \\
V_i &= \frac{1}{\sum_{j=1}^m r_j}\sum_{j=1}^{m} r_j(\bar{y}_{i,j} - \bar{y})^2 \\
V &= \frac{1}{\sum_{j=1}^m r_j}\sum_{j=1}^{m}\sum_{k=1}^{r_j} (y_{i,j}^k - \bar{y})^2
\end{aligned}
$$

S3.3 Monte Carlo filtering

Classification of the input-output curves corresponding to the prediction (the CaMKIIact-PP2Bact balance) The output of the prediction $\boldsymbol{y}^{\text{pred}}$ depends on the input frequency f of the Ca-trains, i.e. $\boldsymbol{y}^{\text{pred}} = \boldsymbol{y}_5^{\text{norm}}(f)$ (see Section S1.3). The different output vectors $\boldsymbol{y}^{\text{pred}}$, corresponding to different parameters $\boldsymbol{\theta}$, were classified into one of two classes based on whether the following constraint was fulfilled or not:

$$
\begin{aligned}
\min_f(y_5^{\text{norm}}(f)) &< y_5^{\text{norm}}(f=0) - 0.1 \\
y_5^{\text{norm}}(f = f_{max}) &> y_5^{\text{norm}}(f=0) + 0.1,
\end{aligned}
$$

where $f = [0:1:20]$ and $f_{\text{max}} = 20$. If the constraint was fulfilled the vector (and the corresponding parameter set $\boldsymbol{\theta}$) were classified as LTD-LTP, otherwise as non-LTD-LTP.

S4 Characteristics of the posterior distribution

A summary of the characteristics of the marginal posterior distributions is given in Table S4 for the free parameters and for the thermo-constrained parameters in Table S5. Histograms of the marginal distributions are given in Figure S8.

S5 Mechanistic understanding of sensitive parameters

We here describe, from a mechanistic point of view, why the the parameters that show up as most sensitive in the Monte Carlo filtering sensitivity analysis have such a large impact on the behaviour in interest.

For the output illustrated in Figure 7 (monotoic versus non-monotonic behavior) the highest scoring parameters correspond to K_d*CaMKII_CaM_Ca3*Ca and K_d*pCaMKII_CaM*Ca (Figure S9), i.e. the K_d values of reactions 22 and 28 in Figure 3. To understand the effect of these parameters we show in Figure S6 the uncertainty of all relevant species of the model during this experimental context (phenotype 5). To ease understanding, the graphs of Figure S6 are sorted in the same way as the model species of Figure 3. In Figure S6 it can be seen that there is a large difference in the species behaviours

Table S4. Arithmetic mean, credibility interval and default parameter value on a log10-scale for the free K_d-parameters and kautMax based on the marginal posterior distributions.

Parameter	Mean	Credibility interval (95%)	Default value	Bimodal
K_d*CaM_Ca3*Ca	3.5	(1.9 , 4.5)	4.2	No
K_d*CaM_Ca2*Ca	5.1	(3.9 , 6.8)	4.4	No
K_d*CaM_Ca1*Ca	1.9	(0.5 , 3.5)	3.1	No
K_d*CaM*Ca	4.9	(3.4 , 6.3)	3.7	No
K_d*CaM_Ca4*PP2B	-1.6	(-1.9 , -1.3)	-1.6	No
K_d*PP2B_CaM_Ca3*Ca	1.0	(-0.3 , 2.1)	1.8	No
K_d*PP2B_CaM_Ca2*Ca	3.1	(1.1 , 4.7)	2.9	No
K_d*PP2B_CaM_Ca1*Ca	2.3	(-0.3 , 4.0)	1.8	Yes
K_d*PP2B_CaM*Ca	2.9	(0.7 , 5.1)	2.8	Yes
K_d*CaM_Ca4*CaMKII	0.8	(-1.0 , 1.7)	1.7	Yes
K_d*CaMKII_CaM_Ca3*Ca	1.8	(0.4 , 3.7)	3.3	No
K_d*CaMKII_CaM_Ca2*Ca	2.5	(0.8 , 5.5)	3.6	No
K_d*CaMKII_CaM_Ca1*Ca	3.2	(0.9 , 6.3)	3.7	No
K_d*CaMKII_CaM*Ca	4.0	(0.8 , 6.2)	3.4	No
K_d*pCaMKII_Ca3*Ca	1.6	(0.4 , 3.2)	3.3	No
K_d*pCaMKII_Ca2*Ca	4.1	(1.7 , 6.0)	3.6	No
K_d*pCaMKII_Ca1*Ca	5.1	(2.4 , 6.4)	3.7	No
K_d*pCaMKII_Ca0*Ca	2.7	(0.6 , 5.6)	3.4	No
K_d*CaM_Ca4*pCaMKIIaut	-0.7	(-2.9 , 1.6)	-0.1	No
kautMax	0.0	(-1.3 , 4.4)	1.7	Yes

Table S5. Arithmetic mean, credibility interval and default parameter value for the thermo-constrained K_d-parameters based on the marginal posterior distributions and rules applied within our model.

Parameter	Mean	Credibility interval (95%)	Default value
K_d*CaM_Ca3*PP2B	0.9	(-1.1 , 2.8)	0.8
K_d*CaM_Ca2*PP2B	2.8	(1.5 , 5.0)	2.3
K_d*CaM_Ca1*PP2B	2.4	(-0.4 , 5.4)	3.5
K_d*CaM*PP2B	4.4	(4.1 , 4.7)	4.4
K_d*CaM_Ca3*CaMKII	2.5	(-0.3 , 4.8)	2.6
K_d*CaM_Ca2*CaMKII	5.1	(2.1 , 7.8)	3.4
K_d*CaM_Ca1*CaMKII	3.7	(-0.5 , 7.7)	2.7
K_d*CaM*CaMKII	4.6	(-0.4 , 9.6)	3.0
K_d*CaM_Ca3*pCaMKIIaut	1.2	(-1.9 , 4.5)	0.8
K_d*CaM_Ca2*pCaMKIIaut	2.2	(-0.7 , 5.6)	1.5
K_d*CaM_Ca1*pCaMKIIaut	-1.0	(-4.2 , 3.1)	0.9
K_d*CaM*pCaMKIIaut	1.2	(-1.6 , 4.3)	1.2

between the monotonic and non-monotonic case, especially for [pCaMKII] and [CaMKII_CaM_Ca3] and that this can explain the non-monotounic dip in the output illustrated in Figure 7 (i.e. dip in Mol Ca bounded per Mol CaM at [Ca] levels between 3<log([Ca])<3.5). In this region [pCaMKII] (which has no Ca bound) increases a lot when the non-monotonic case is considered, whereas [CaMKII_CaM_Ca3] (which has three Ca bound) decreases from a high level. Such a behavior should be enforced by a high K_d of reaction 22 as well as reaction 28, and this is exactly what the histograms of the top scoring parameters show.

When it comes to the LTD/LTP switch the explanation is easier. Comparing the top panels of Figure 8 it can be seen that an important difference between class LTD/LTP and non-LTD/LTP is that the CaMKIIact-PP2Bact balance is negative for low Ca-frequencies of class LTD/LTP, i.e. there is a lot of active PP2B (i.e. [PP2B_CaM_Ca4]) at small frequencies. The parameter shown to be most important in separating between the classes (kf*PP2B_CaM_Ca3*Ca, see Figure S10) are part of the reaction leading directly to more active PP2B. This parameter corresponds to the binding of the fourth Calcium ion to PP2B_CaM. When the forward rate of this reaction is high (as for class LTD/LTP in Figure S10) this will result in more PP2B_CaM_Ca4 being formed.

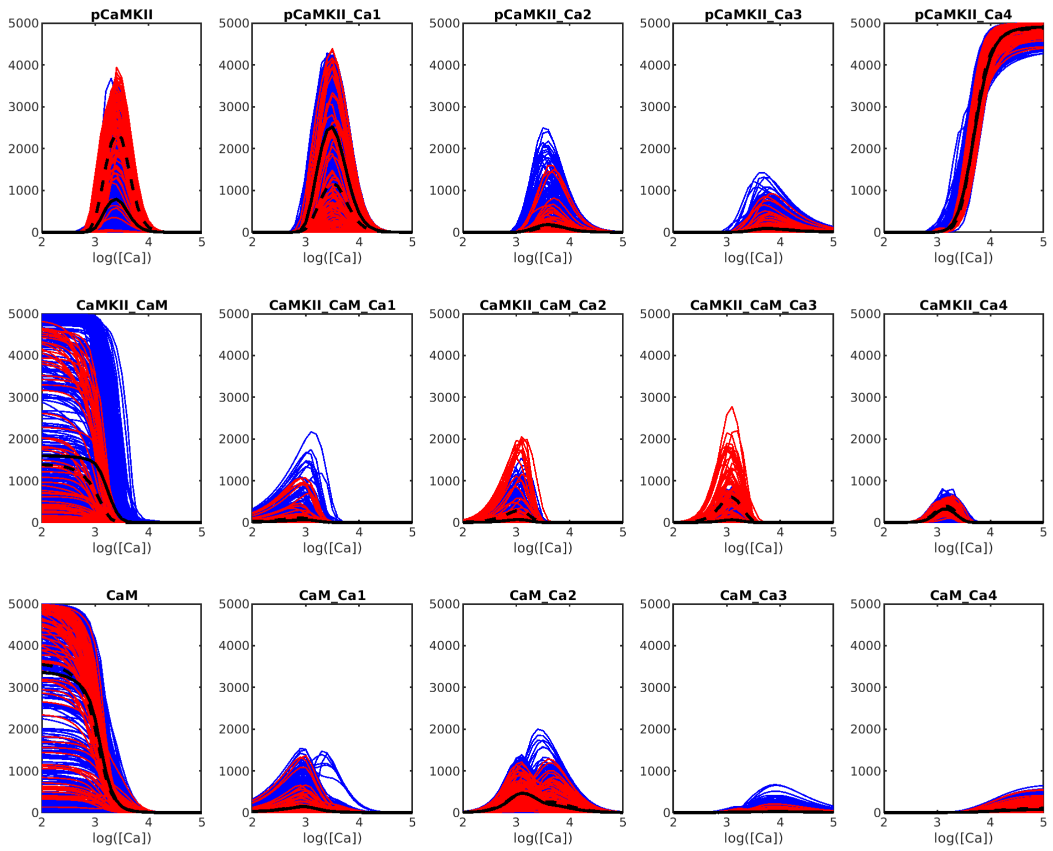

Fig. S6. Uncertainty in the species concentrations, $\boldsymbol{x}(t = 600)$, for different [Ca] levels using the experimental setting of phenotype 5. The dashed line corresponds to the average behavior for the non-monotonic class, the solid line to the average behaviour for the monotonic class. The graphs are sorted in the same order as the corresponding species of Figure 3. Concentrations are in nM.

S6 Retrospective restriction of the posterior sample

The posterior sample was filtered based on experimental ranges from literature to see the effect on the prediction uncertainty as well as the parameter sensitivity. This was done by rejection sampling from the copula of the posterior sample, using the new restrictions. The results are shown in Figure S7. As expected, restriction based on the parameter ranges of Table 1 in Stefan *et al.*, 2008 does neither decrease the uncertainty nor change the sensitivity much as compared to the original analysis (compare Figure S7 left and Figure 6) since the parameters that are restricted do not contribute much to the uncertainty of the prediction in the first place (Figure 6). However, when one of the most sensitive parameters from the first analysis $\mathrm{K_d}$*CaM*CaMKII (Figure 6) is restricted according to the range given in the Supplementary table of Pepke *et al.*, 2010 there is a large change in the sensitivity (Figure S7 right), and all parameters that were part of the cluster containing $\mathrm{K_d}$*CaM*CaMKII (Figure 5) have a large reduction in sensitivity (Figure S7 right).

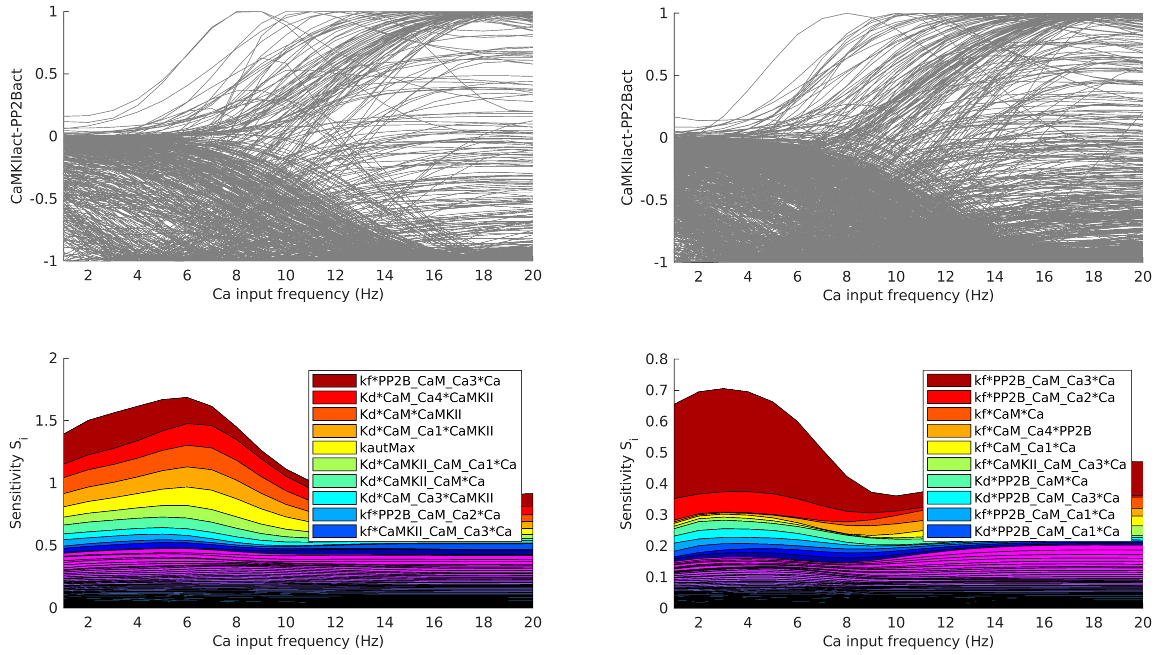

Fig. S7. Sensitivity analysis of the retrospectively restricted posterior sample. Left: Restriction based on the parameter ranges of Table 1 in Stefan et al., 2008. Right: Restriction based on the parameter values corresponding to K_d*CaM*CaMKII in the Supplementary table of Pepke et al., 2010.

S7 Analytical equilibrium model reduction

Analytical solutions were obtained for phenotypes 1-4 (cf. Table S2) as follows: We note that these subsystems only consist of reversible reactions of the form $A + B \rightleftharpoons C$, and hence all the reaction fluxes for these subsystems will be of the form $k_f[A][B] - k_r[C]$. At equilibrium, all the reaction fluxes are zero, and so, we can solve the equations by writing them as $k_f[A][B] = k_r[C]$, and then taking logarithms on both sides, giving $\log[A] + \log[B] - \log[C] = \log(K_d)$. Note that these equations are linear in the logarithm of the species concentrations. The number of such equations is the same as the number of reactions in the subsystem, and note that different species concentrations will come in as $[A]$, $[B]$ and $[C]$ in the equation above. To keep track of which species are the reactants and the product in this system, we make use of the stoichiometry matrix. We number the species and reactions of the subsystem that we are considering, according to some order, and ignore other reactions and species that do not occur in this subsystem. We denote the unknown equilibrium concentrations by the column vector X, and the equilibrium constants by the column vector K_d. The entries of the K_d vector will in this section be denoted by Kd1, Kd2, ..., where the number of the parameter coincides with the ID of the particular reaction (as given in Table S2). The stoichiometry matrix N is defined by its entries

$$N_{ij} = \begin{cases} -1 & \text{if species } i \text{ is one of the reactants of reaction } j, \\ 1 & \text{if species } i \text{ is the product of reaction } j, \\ 0 & \text{otherwise.} \end{cases}$$

The system of equations that we need to solve can then be written in matrix form as

$$-N^T \log(X) = \log(K_d), \tag{S5}$$

where $\log(X)$, $\log K_d$ denotes the column vector consisting of the logarithms of the entries of X, K_d, respectively. This system has nontrivial solutions if and only if the vector $\log(K_d) \in \mathrm{Ran}(N^T) = (\ker N)^{\perp}$, where $\mathrm{Ran}(N^T)$ and $\ker(N)$ denote the range of N^T and kernel (null space) of N, respectively. Here, $\perp$ denotes the orthogonal complement. The general solution of this system is found as the sum of the general solution to the corresponding homogeneous equation and a particular solution of the inhomogeneous system. Thus, the number of free parameters in the general solution is the same as the dimension of the null space of N^T. Uniqueness of solutions will only be obtained when the conservation laws are taken into account. These conservation laws can also be determined from the kernel of N^T. The conservation laws are of the form $C^T X =$ total amount, where C^T is a fixed row vector of nonnegative integers, of the same length as the number of species in the subsystem. The vector C can be found as a basis vector of the null space of N^T. Now we will find the explicit solutions for the subsystems.

S7.1 Explicit solutions for the subsystem of phenotype 1

The species that are present in this subsystem are CaM, CaM_Ca1, CaM_Ca2, CaM_Ca3, CaM_Ca4. We denote the equilibrium concentrations of these species with $X_1, \ldots, X_5$ (with the same order as above). The stoichiometry matrix N is given by

$$N = \begin{pmatrix} -1 & 0 & 0 & 0 \\ 1 & -1 & 0 & 0 \\ 0 & 1 & -1 & 0 \\ 0 & 0 & 1 & -1 \\ 0 & 0 & 0 & 1 \end{pmatrix}.$$

The system (S5) has nontrivial solutions for all values of the K_d parameters, since $\ker(N) = \{0\}$. A basis for the null space of N^T is $C_1 := \{(1, 1, 1, 1, 1)^T\}$. As a particular solution of the system (S5), we take

$$X_p = \left(\frac{\text{Kd1} \cdot \text{Kd2} \cdot \text{Kd3} \cdot \text{Kd4}}{\text{Ca}^4}, \frac{\text{Kd2} \cdot \text{Kd3} \cdot \text{Kd4}}{\text{Ca}^3}, \frac{\text{Kd3} \cdot \text{Kd4}}{\text{Ca}^2}, \frac{\text{Kd4}}{\text{Ca}}, 1 \right)^T. \tag{S6}$$

Since the nullspace of N^T is one-dimensional, and spanned by C_1,we obtain the general solution of (S5) as

$$X = \alpha^{C_1} \star X_p = \alpha X_p, \tag{S7}$$

where $\star$ denotes the Hadamard (i.e. pointwise) product and α^{C_1} denotes the vector which is formed by raising the number α to each separate entry of C_1. To determine α, we invoke the conservation law $C_1^T X =$ totalCaM, and obtain

$$\alpha(C_1^T X_p) = \text{totalCaM}.$$

Solving this equation for α, and substituting the obtained expression for α into (S7), we obtain the equilibrium solution

$$X = \frac{\text{totalCaM}}{C_1^T X_p} X_p.$$

Finally, we compute the output for phenotype 1 as

$$\text{MolCaPerMolCaM} = \frac{(0, 1, 2, 3, 4)X}{\text{totalCaM}} = \frac{(0, 1, 2, 3, 4)X_p}{(1, 1, 1, 1, 1)X_p} \tag{S8}$$

with X_p as in (S6).

S7.2 Phenotype 2

Only reaction 5 is active in this subsystem, and the equilibrium concentrations are [CaM] $=: X_1$, [PP2B] $=: X_2$, and [PP2B_CaM] $=: X_3$. The stoichiometry matrix is $N = (-1, -1, 1)^T$. The system (S5) has nontrivial solutions for all values of K_d since $\ker(N) = \{0\}$. We find a particular solution (S5) as

$$X_p := (1, 1, \text{Kd5})^T.$$

The kernel of N^T is spanned by $C_1 := (1, 0, 1)^T$ and $C_2 := (0, 1, 1)^T$. There are two conservation laws: $C_1^T X = \text{totalCaM}$ and $C_2^T X = \text{totalPP2B}$. The general equilibrium solution is therefore of the form

$$X = \alpha^{C_1} \star \beta^{C_2} \star X_p = \begin{pmatrix} \alpha \\ \beta \\ \alpha\beta\text{Kd5} \end{pmatrix},$$

and from the conserved quantities we obtain the system of equations

$$\begin{cases} \alpha + \alpha\beta\text{Kd5} = \text{totalCaM}, \\ \beta + \alpha\beta\text{Kd5} = \text{totalPP2B}. \end{cases} \tag{S9}$$

This system is reduced to a quadratic equation in α (by solving the second equation for β and substituting the solution into the first equation). The quadratic equation has one positive and one negative root, and since $\alpha = [\text{CaM}]$ cannot be negative, only the positive root is relevant. This way we obtain

$$\begin{aligned} \alpha = &-\frac{1}{2}\left(\frac{1}{\text{Kd5}} + \text{totalPP2B} - \text{totalCaM}\right) \\ &+ \sqrt{\frac{1}{4}\left(\frac{1}{\text{Kd5}} + \text{totalPP2B} - \text{totalCaM}\right)^2 + \text{totalCaM}}. \end{aligned} \tag{S10}$$

Finally, the output MolCaMPerMolPP2B is computed as

$$\begin{aligned} \text{MolCaMPerMolPP2B} &= \frac{[\text{PP2B\_CaM}]}{[\text{PP2B}] + [\text{PP2B\_CaM}]} = \frac{(0,0,1)X}{(0,1,1)X} \\ &= \frac{\alpha\text{Kd5}}{1 + \alpha\text{Kd5}}, \end{aligned} \tag{S11}$$

with α as in (S10).

S7.3 Phenotypes 3 and 4

The subsystems of phenotype 3 and 4 are the same. The only difference is the total amount of CaM. The subsystem has 11 species, 13 reactions and 2 conservation laws. The computations are very similar to those of phenotype 2, although the formulas are longer. The species concentrations are denoted by $X_1 :=$ [CaM], $X_2 :=$ [CaM_Ca1], $X_3 :=$ [CaM_Ca2], $X_4 :=$ [CaM_Ca3], $X_5 :=$ [CaM_Ca4], $X_6 :=$ [PP2B], $X_7 :=$ [PP2B_CaM], $X_8 :=$ [PP2B_CaM_Ca1], $X_9 :=$ [PP2B_CaM_Ca2], $X_{10} :=$ [PP2B_CaM_Ca3], $X_{11} :=$ [PP2B_CaM_Ca4]. The nullspace of N has dimension 4, and it is spanned by

$$\begin{aligned} W_1 &:= (-1, 0, 0, 0, 1, -1, 0, 0, 0, 1, 0, 0, 0)^T, \\ W_2 &:= (0, -1, 0, 0, 0, 1, -1, 0, 0, 0, 1, 0, 0)^T, \\ W_3 &:= (0, 0, -1, 0, 0, 0, 1, -1, 0, 0, 0, 1, 0)^T, \\ W_4 &:= (0, 0, 0, -1, 0, 0, 0, 1, -1, 0, 0, 0, 1)^T. \end{aligned}$$

The system (S5) has a nontrivial solution if and only if $\log(K_d) \in \ker(N)^\perp$, i.e. if and only if $\log(K_d)$ is orthogonal to W_j, $j = 1, \ldots, 4$. These conditions read

$$\begin{cases} -\log(\text{Kd1}) + \log(\text{Kd5}) - \log(\text{Kd6}) + \log(\text{Kd10}) = 0, \\ -\log(\text{Kd2}) + \log(\text{Kd6}) - \log(\text{Kd7}) + \log(\text{Kd11}) = 0, \\ -\log(\text{Kd3}) + \log(\text{Kd7}) - \log(\text{Kd8}) + \log(\text{Kd12}) = 0, \\ -\log(\text{Kd4}) + \log(\text{Kd8}) - \log(\text{Kd9}) + \log(\text{Kd13}) = 0, \end{cases} \tag{S12}$$

which is equivalent to the Wegscheider conditions for this subsystem (cf. Table S3).

The null space of N^T has dimension 2, and it is spanned by

$$\begin{aligned} C_1 &:= (1,1,1,1,1,0,1,1,1,1,1)^T, \\ C_2 &:= (0,0,0,0,0,1,1,1,1,1,1)^T, \end{aligned} \tag{S13}$$

giving rise to the conservation laws $C_1X = \text{totalCaM}$ and $C_2X = \text{totalPP2B}$. A particular solution of (S5) is given by

$$X_p = \begin{pmatrix} \frac{\text{Kd1}\cdot\text{Kd2}\cdot\text{Kd3}\cdot\text{Kd4}\cdot\text{Kd9}}{\text{Ca}^4} \\ \frac{\text{Kd2}\cdot\text{Kd3}\cdot\text{Kd4}\cdot\text{Kd9}}{\text{Ca}^3} \\ \frac{\text{Kd3}\cdot\text{Kd4}\cdot\text{Kd9}}{\text{Ca}^2} \\ \frac{\text{Kd4}\cdot\text{Kd9}}{\text{Ca}} \\ \text{Kd9} \\ 1 \\ \frac{\text{Kd1}\cdot\text{Kd2}\cdot\text{Kd3}\cdot\text{Kd4}\cdot\text{Kd9}}{\text{Ca}^4\cdot\text{Kd5}} \\ \frac{\text{Kd2}\cdot\text{Kd3}\cdot\text{Kd4}\cdot\text{Kd9}}{\text{Ca}^3\cdot\text{Kd6}} \\ \frac{\text{Kd3}\cdot\text{Kd4}\cdot\text{Kd9}}{\text{Ca}^2\cdot\text{Kd7}} \\ \frac{\text{Kd4}\cdot\text{Kd9}}{\text{Ca}\cdot\text{Kd8}} \\ 1 \end{pmatrix}, \tag{S14}$$

and the general solution is given by

$$X = \alpha^{C_1} \star \beta^{C_2} \star X_p,$$

Where C_1 and C_2 are given by (S13) and X_p is as in (S14). The conservation laws give rise to the system

$$\begin{cases} C_1^T(\alpha^{C_1} \star \beta^{C_2} \star X_p) = \text{totalCaM}, \\ C_2^T(\alpha^{C_1} \star \beta^{C_2} \star X_p) = \text{totalPP2B}, \end{cases}$$

or equivalently,

$$\begin{cases} \alpha\beta(C_1 \star C_2)^T X_p + \alpha(C_1 \star (\mathbb{1} - C_2))^T X_p = \text{totalCaM}, \\ \alpha\beta(C_1 \star C_2)^T X_p + \beta(C_2 \star (\mathbb{1} - C_1))^T X_p = \text{totalPP2B}, \end{cases} \tag{S15}$$

where $\mathbb{1}$ is a column vector of length 11 where all entries are 1. The system (S15) can be simplified further by noting that $(C_2 \star (\mathbb{1} - C_1))^T X_p = 1$, and can be solved in the same way as (S9), leading to the quadratic equation

$$\alpha^2 + \alpha\left(\frac{1}{(C_1 \star C_2)^T X_p} + \frac{\text{totalPP2B} - \text{totalCaM}}{(C_1 \star (\mathbb{1} - C_2))^T X_p}\right) - \frac{\text{totalCaM}}{((C_1 \star C_2)^T X_p)(C_1 \star (\mathbb{1} - C_2))^T X_p} = 0$$

in α with one positive and one negative root. Again, it is only the positive root that is valid, and so

$$\alpha = -\frac{1}{2}\left(\frac{1}{(C_1 \star C_2)^T X_p} + \frac{\text{totalPP2B} - \text{totalCaM}}{(C_1 \star (\mathbb{1} - C_2))^T X_p}\right) + \sqrt{\frac{1}{4}\left(\frac{1}{(C_1 \star C_2)^T X_p} + \frac{\text{totalPP2B} - \text{totalCaM}}{(C_1 \star (\mathbb{1} - C_2))^T X_p}\right)^2 + \frac{\text{totalCaM}}{((C_1 \star C_2)^T X_p)(C_1 \star (\mathbb{1} - C_2))^T X_p}}. \tag{S16}$$

The output for both phenotypes 3 and 4 is

$$\text{activePP2BPercentage} = 100\frac{X_{11}}{\text{totalPP2B}} = 100\frac{\alpha}{1 + \alpha(C_1 \star C_2)^T X_p}, \tag{S17}$$

with α given by (S16), and $(C_1 \star C_2)^T X_p$ is the sum of the last five entries of X_p, with X_p given by (S14).

S7.4 Phenotypes 5 and 6

Since the subsystems contains a reaction which is neither elementary nor irreversible, the method that was used in Sections S7.1–S7.3 is not applicable. For this reason, we have simulated the ODE systems instead of using analytical equilibrium solutions when computing these outputs.

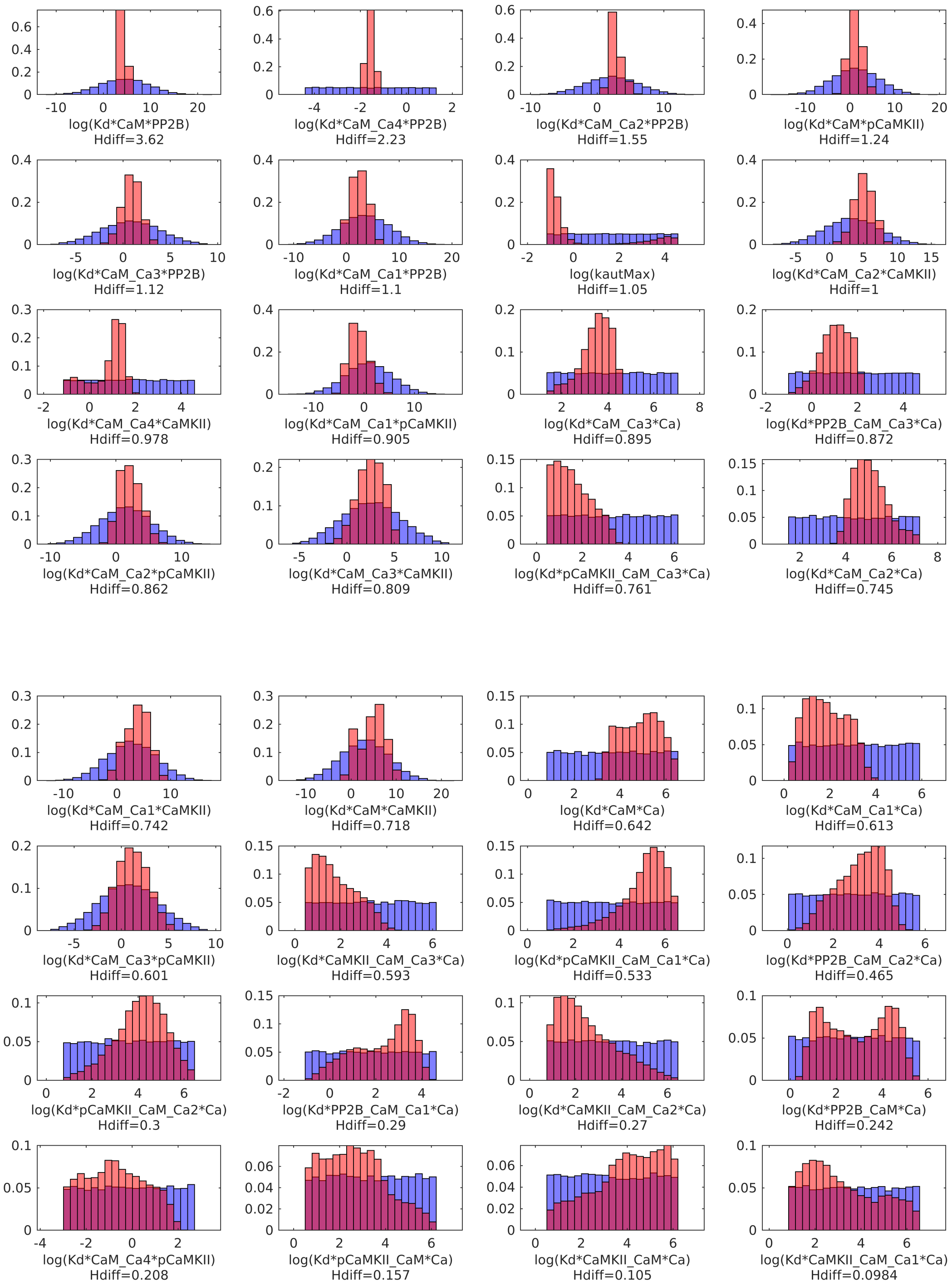

Fig. S8. Marginal prior and posterior distributions for the model parameters sorted by reduction in entropy (H_{diff}). Normalized sample histograms from the prior (blue) and posterior (red) distributions of the free and thermodynamically constrained K_d parameters of the model. The prior of the free parameters correspond to a sample from a log-uniform distribution centered around the default parameter values, while the priors of the thermodynamically constrained parameters are the same samples transformed by the constraint rules given in Table S3.

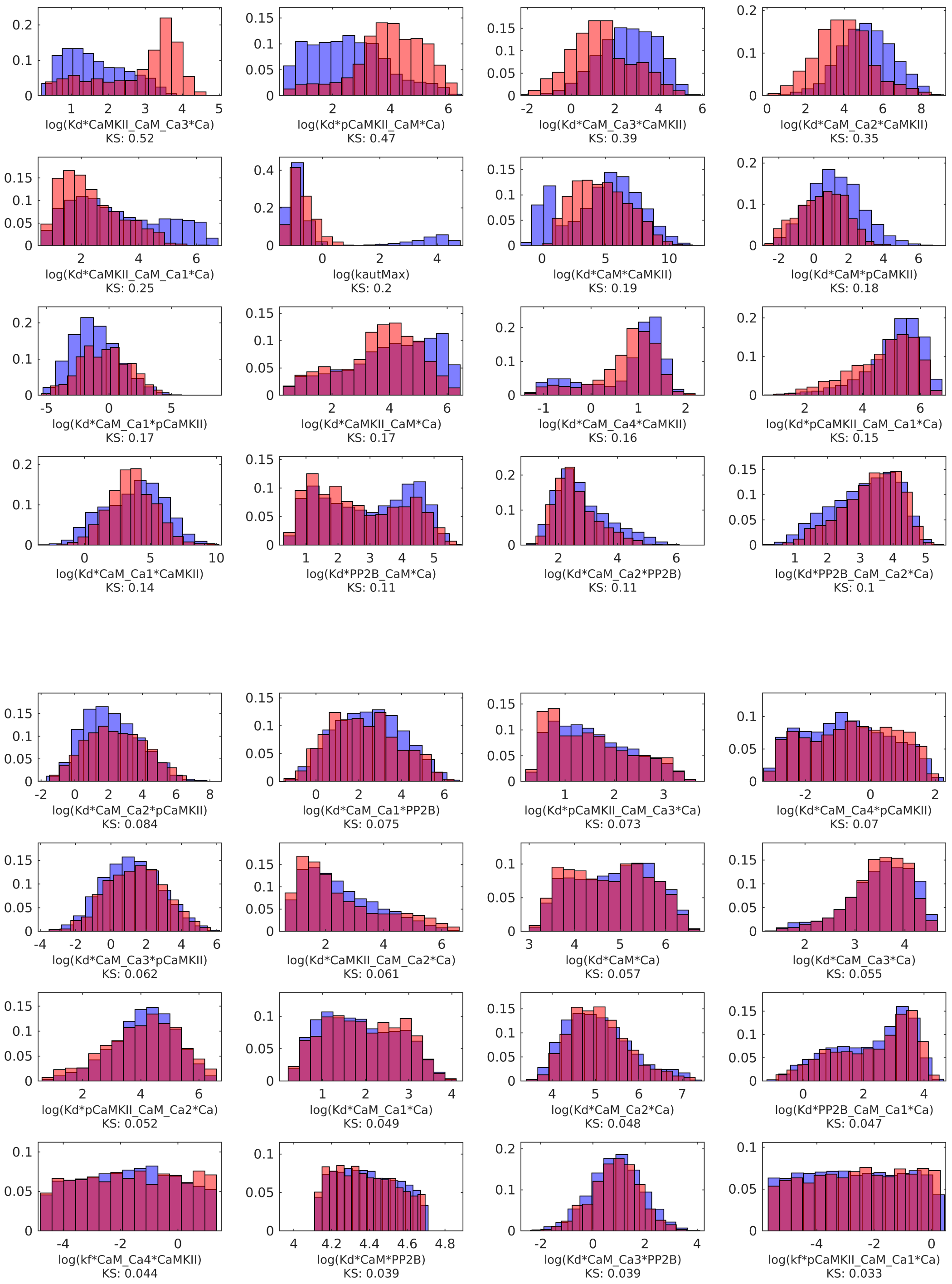

Fig. S9. Normalized histograms describing the marginal posterior distributions of the model parameters subdivided into two classes depending on the behaviour of the output function corresponding to phenotype 5. The colors correspond to the classes defined in Figure 7; blue=monotonic (Figure 7, top left), red=non-monotonic (Figure 7, top right). The parameter histograms are sorted according to the Kolmogorov-Smirnov test statistica.

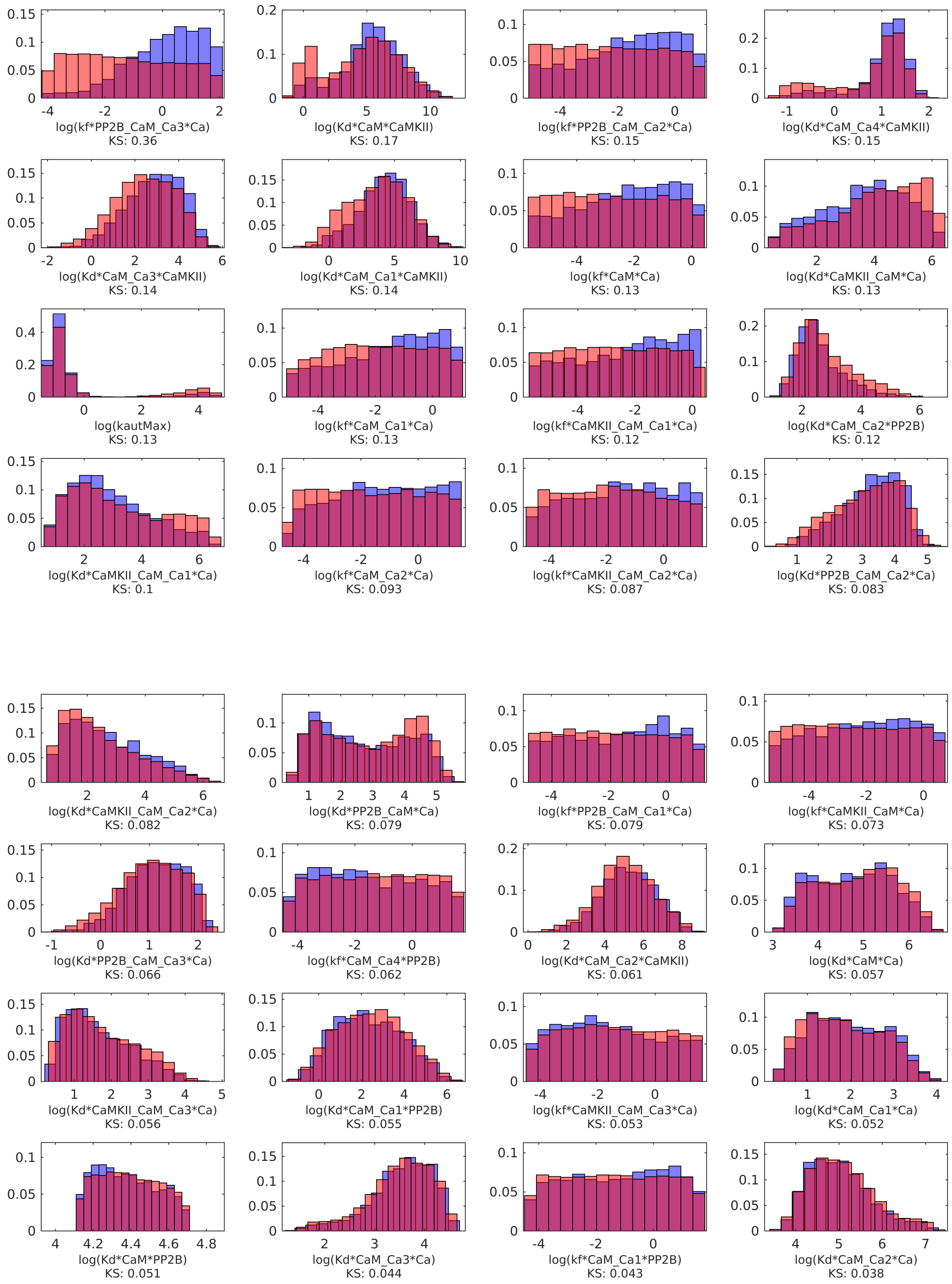

Fig. S10. Normalized histograms describing the marginal posterior distributions of the model parameters subdivided into two classes depending on the behaviour of the output function corresponding to the prediction. The colors correspond to the classes defined in Figure 8; blue=LTD-LTP (Figure 8, top left), red=non-LTD-LTP (Figure 8, top right). The parameter histograms are sorted according to the Kolmogorov-Smirnov test statistica.

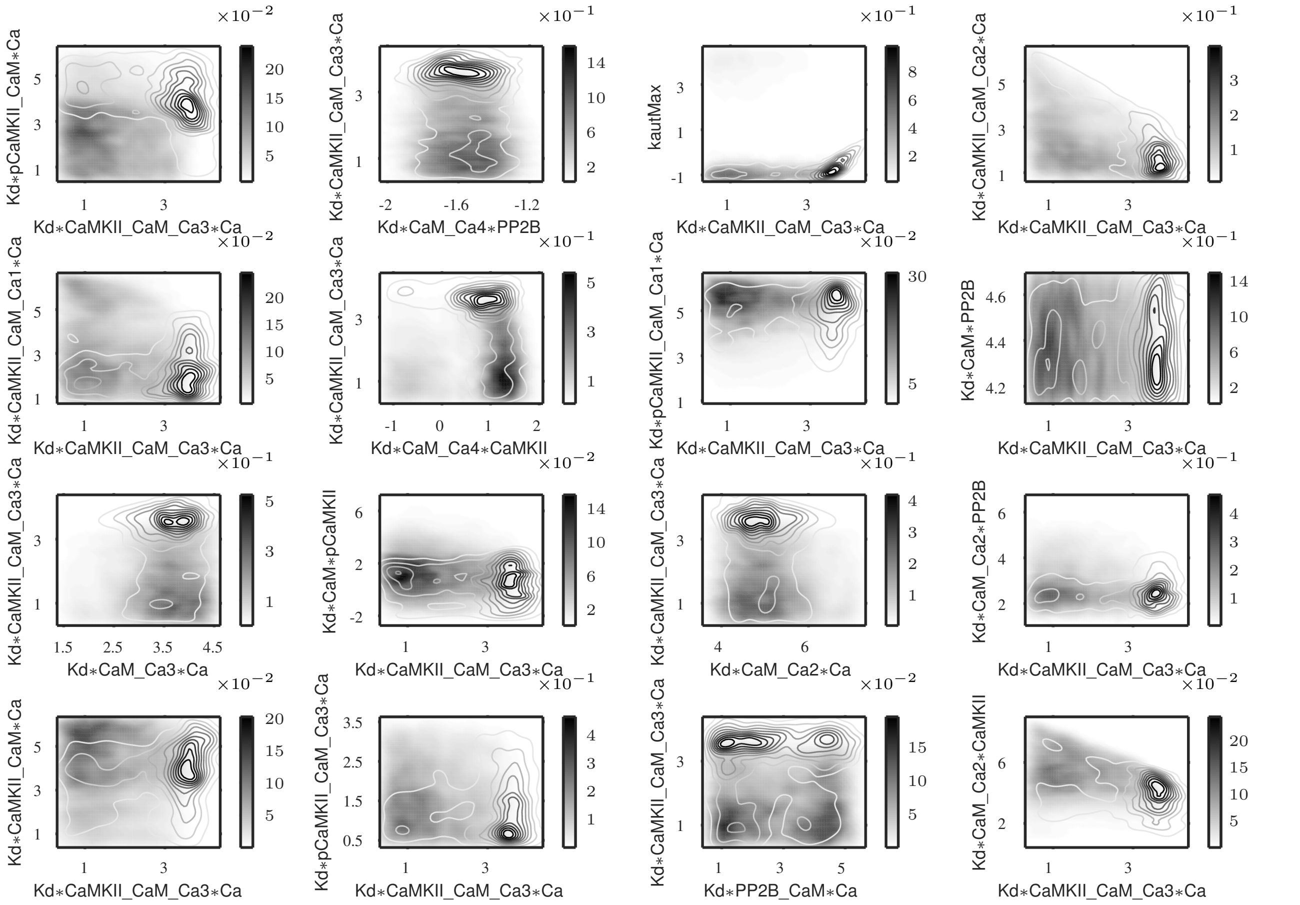

Fig. S11. The outputs corresponding to phenotype 5 were divided into two classes, monotonic behaviour of the observed output function and non-monotonic behaviour. The subsamples corresponding to these two classes were further investigated via pairwise projections to find the pairs that cause these different behaviours. The density of parameters resulting in monotonic output behavior are represented by color shading and the parameters resulting in non-monotonic behaviour are shown as contour plots using the same color scheme. That way the lines are only visible if there is a difference in the two projected densities. If the densities are very different then the parameters pair is important for this classification. We calculated the Kullback Leibler Divergence (KLD) for each pair; this picture shows the top 16 pairs with highest KLD values.

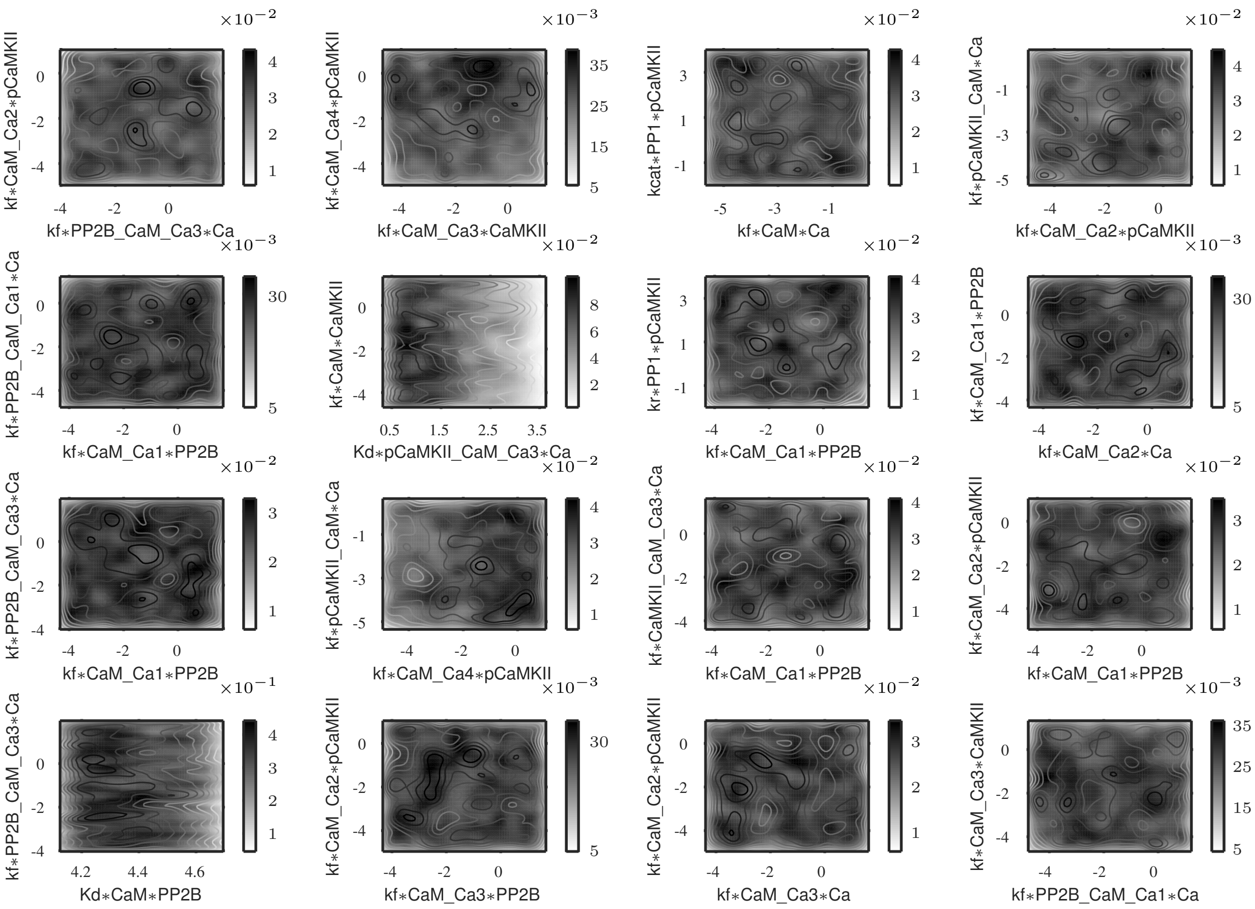

Fig. S12. For illustration purposes, this figure shows the corresponding bottom 16 parameter pairs, ranked by KLD score. The figure was produced in the same fashion as Figure S11. The contour lines depicting parameters with resulting non-monotonic output behaviour and the color shading (for monotonic behaviour) cannot be distinguished because the densities are so similar.

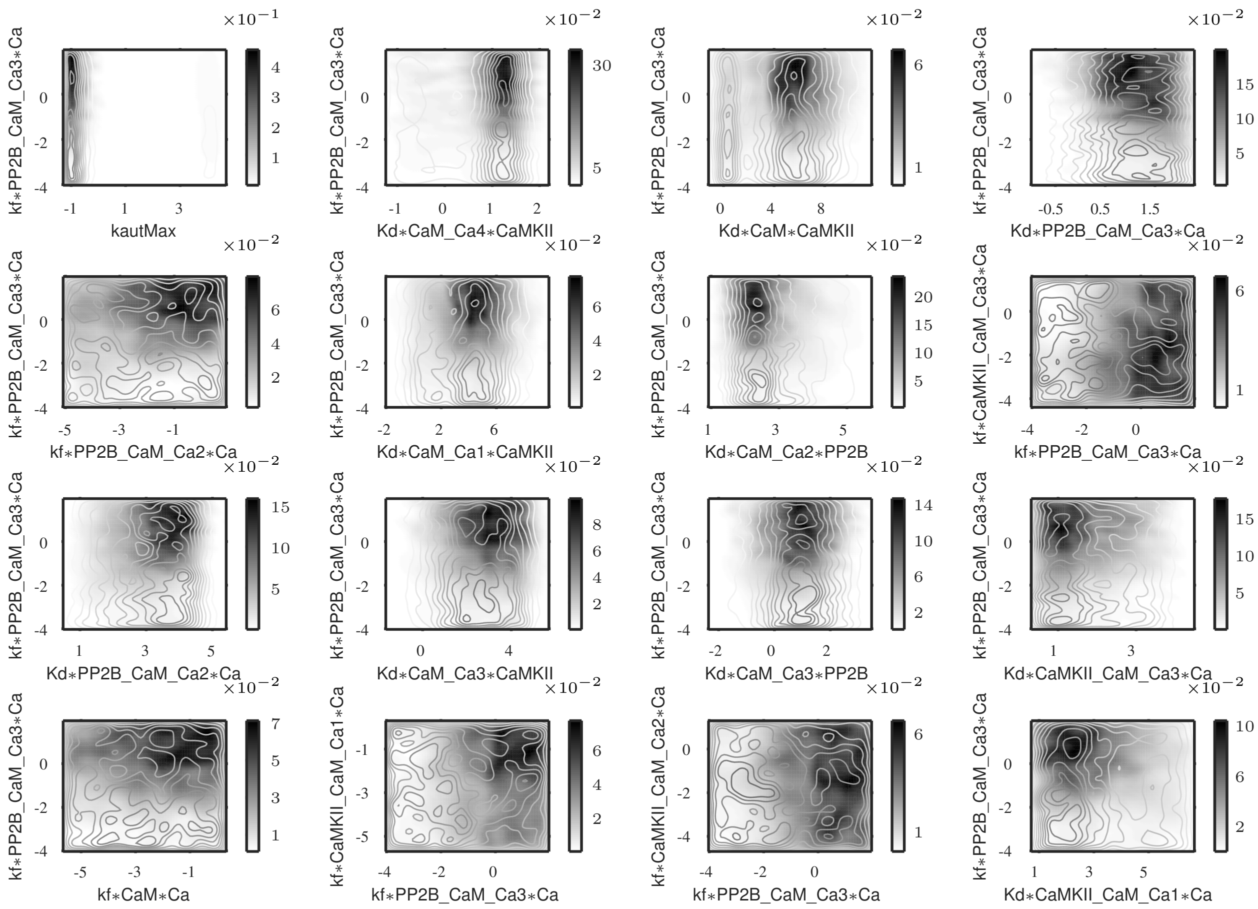

Fig. S13. The outputs corresponding to the prediction were divided into two classes: LTD-LTP or non-LTD-LTP as described in the main text. The subsamples corresponding to these two classes were further investigated via pairwise projections to find the pairs that cause these different behaviours: color shading shows the density responsible for output behaviour LTD-LTP, while the contour lines indicate the subsamples density of non-LTD-LTP. If the two projected probability densities are very different then the parameter pair is important for this classification, in such cases the lines can be seen clearly, they are colored using the same colormap. We calculated the Kullback Leibler Divergence (KLD) for each pair; this picture shows the top 16 pairs with highest KLD values.

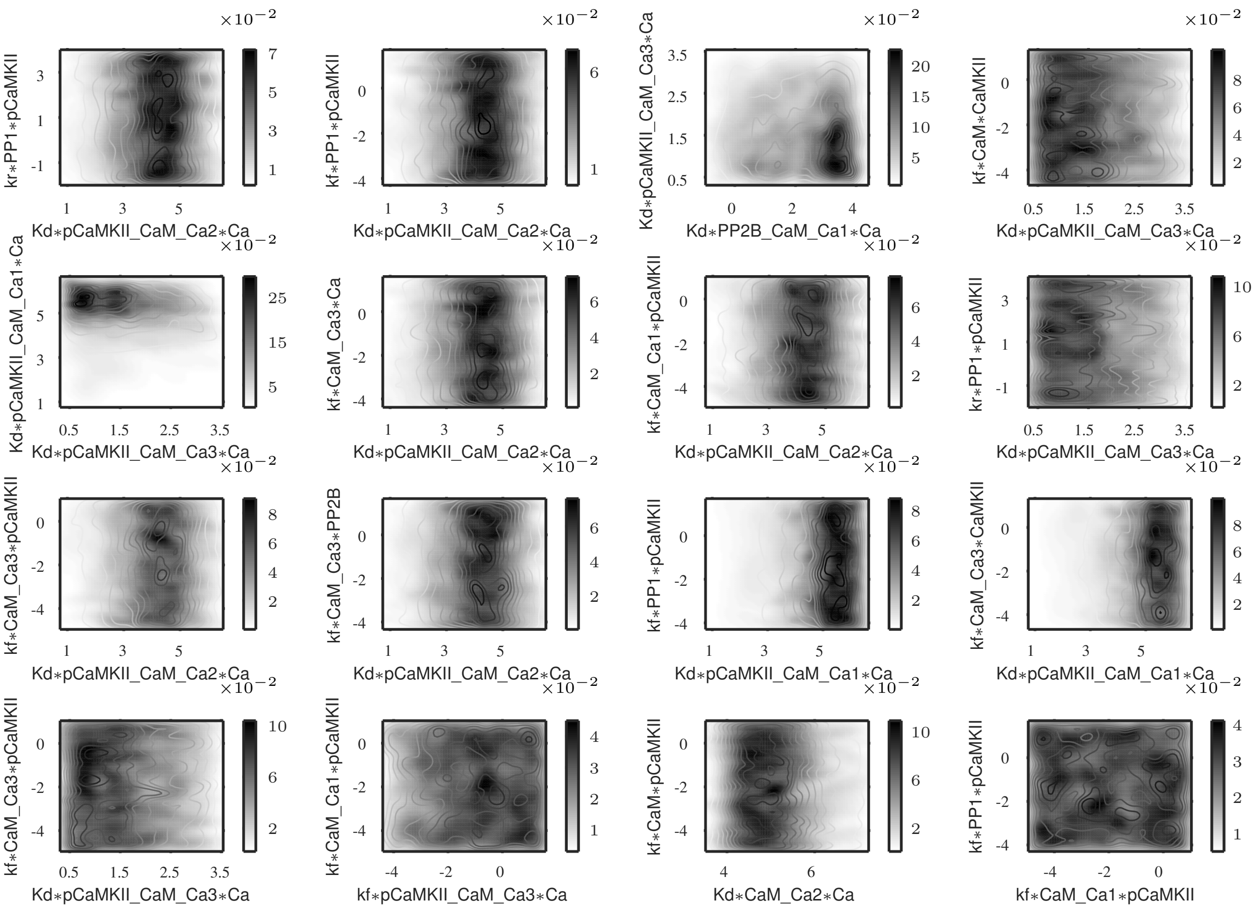

Fig. S14. For illustration purposes, this figure shows the corresponding bottom 16 parameter pairs, with lowest KLD values. It was produced in the same fashion as Figure S13 and shows no discernible difference between the two classes for each parameter pair. The color shading indicates the density of parameter vectors of class LTD-LTP while the contour lines represent the complement of that sample (parameters belonging to class non-LTD-LTP).

References

Aas, K., Czado, C., Frigessi, A., and Bakken, H. (2009). Pair-copula constructions of multiple dependence. *Insurance: Mathematics and Economics*, **44**(2), 182 – 198.

Bedford, T. and Cooke, R. M. (2002). Vines–a new graphical model for dependent random variables. *Ann. Statist.*, **30**(4), 1031–1068.

Brechmann, E. C. and Schepsmeier, U. (2013). Modeling dependence with c- and d-vine copulas: The R package CDVine. *Journal of Statistical Software*, **52**(3), 1–27.

Li, L., Stefan, M. I., and Le Novère, N. (2012). Calcium input frequency, duration and amplitude differentially modulate the relative activation of calcineurin and camkii. *PloS one*, **7**(9), e43810.

Nair, A. G., Gutierrez-Arenas, O., Eriksson, O., Jauhiainen, A., Blackwell, K. T., and Kotaleski, J. H. (2014). Chapter twelve - modeling intracellular signaling underlying striatal function in health and disease. In K. T. Blackwell, editor, *Computational Neuroscience*, volume 123 of *Progress in Molecular Biology and Translational Science*, pages 277–304. Academic Press.

Nelsen, R. (2006). An introduction to copulas, 2nd edition. *New York: SpringerScience Business Media*.

Pepke, S., Kinzer-Ursem, T., Mihalas, S., and Kennedy, M. B. (2010). A dynamic model of interactions of ca2+, calmodulin, and catalytic subunits of ca2+/calmodulin-dependent protein kinase ii. *PLoS computational biology*, **6**(2), e1000675.

Saltelli, A., Tarantola, S., Campolongo, F., and Ratto, M. (2004). *Sensitivity analysis in practice: a guide to assessing scientific models*. John Wiley & Sons.

Schepsmeier, U., Stoeber, J., Brechmann, E. C., Graeler, B., Nagler, T., and Erhardt, T. (2018). *VineCopula: Statistical Inference of Vine Copulas*. R package version 2.1.4.

Stefan, M. I., Edelstein, S. J., and Le Novère, N. (2008). An allosteric model of calmodulin explains differential activation of pp2b and camkii. *Proceedings of the National Academy of Sciences*, **105**(31), 10768–10773.
